# Molecular phylogeny of Squaliformes and first occurrence of bioluminescence in sharks

**DOI:** 10.1186/s12862-015-0446-6

**Published:** 2015-08-16

**Authors:** Nicolas Straube, Chenhong Li, Julien M. Claes, Shannon Corrigan, Gavin J. P. Naylor

**Affiliations:** Friedrich Schiller Universität Jena, Leutragraben 1, 07743 Jena, Germany; Hollings Marine Laboratory, 331 Fort Johnson Rd, Charleston, SC 29412 USA; Bavarian State Collection of Zoology, Münchhausenstraße 21, 81247 Munich, Germany; Key Laboratory of Exploration and Utilization of Aquatic Genetic Resources, Shanghai Ocean University, Ministry of Education, Shanghai, 201306 China; Marine Biology Laboratory, Earth and Life Institute, Université catholique de Louvain, Kellner building, 3 Place Croix du Sud - bte L7.06.04, 1348 Louvain-la-Neuve, Belgium

## Abstract

**Background:**

Squaliform sharks represent approximately 27 % of extant shark diversity, comprising more than 130 species with a predominantly deep-dwelling lifestyle. Many Squaliform species are highly specialized, including some that are bioluminescent, a character that is reported exclusively from Squaliform sharks within Chondrichthyes. The interfamiliar relationships within the order are still not satisfactorily resolved. Herein we estimate the phylogenetic interrelationships of a generic level sampling of “squaloid” sharks and closely related taxa using aligned sequences derived from a targeted gene capture approach. The resulting phylogenetic estimate is further used to evaluate the age of first occurrence of bioluminescence in Squaliformes.

**Results:**

Our dataset comprised 172 putative ortholog exon sequences. Phylogenetic estimates result in a fully resolved tree supporting a monophyletic lineage of Squaliformes excluding *Echinorhinus*. Non-luminous Squalidae are inferred to be the sister to a clade comprising all remaining Squaliform families. Our results suggest that the origin of photophores is coincident with an elevated diversification rate and the splitting of families Dalatiidae, Etmopteridae, Oxynotidae and Somniosidae at the transition of the Lower to the Upper Cretaceous. The presence of luminous organs was confirmed for the Sleeper shark genus *Zameus*. These results indicate that bioluminescence in sharks is not restricted solely to the families Etmopteridae and Dalatiidae as previously believed.

**Conclusions:**

The sister-clade to non-luminous Squalidae comprises five families. The presence of photophores is reported for extant members of three out of these five families based on results of this study, i.e. Lantern sharks (Etmopteridae), Kitefin sharks (Dalatiidae) and Sleeper sharks (Somniosidae). Our results suggest that the origin of luminous organs arose during the rapid diversification event that gave rise to the extant Squaliform families. These inferences are consistent with the idea of diversification of Squaliform sharks being associated with the emergence of new deep-sea habitats in the Lower Cretaceous, which may have been facilitated by the evolution of bioluminescence.

**Electronic supplementary material:**

The online version of this article (doi:10.1186/s12862-015-0446-6) contains supplementary material, which is available to authorized users.

## Background

Squaliform sharks constitute a group of highly specialized species with a predominantly deep-dwelling lifestyle. They represent a substantial part of extant shark diversity (~27 % [1]) comprising 24 genera and more than 130 described species [2]. Many Squaliform species are bioluminescent, a feature which appears to be exclusive within the Chondrichthyes. Currently, the families Echinorhinidae (Bramble - and Prickly sharks), Squalidae (Dogfish sharks), Centrophoridae (Gulper Sharks), Somniosidae (Sleeper sharks), Oxynotidae (Rough sharks), Dalatiidae (Kitefin sharks), and Etmopteridae (Lantern sharks) are discussed to form the Squaliformes. However, some previous morphological studies have suggested alternative intergeneric and interfamilial arrangements for the group [2–12].

The phylogenetic placement of Echinorhinidae has remained ambiguous in both morphological and molecular studies, either being included within Squaliformes, considered sister to Squaliformes, or placed in a separate group with Saw sharks (Pristiophoriformes) or Angel sharks (Squatiniformes). Further, recent molecular studies have recovered Squalidae, Centrophoridae, Dalatiidae, and Etmopteridae as monophyletic lineages within the Squaliformes, however, their interfamiliar relationships remain partially unresolved while the family Somniosidae appeared paraphyletic as Oxynotidae cluster within Somniosidae [2, 3, 8–24].

All of the molecular data sets examined to date have been based on the analysis of a single or few genes and none have recovered substantial support for branching events at the family level, likely due to limited phylogenetic signal supporting deeper nodes. Phylogenetic analyses based on morphological characters have not yielded consistent results either, e.g. [9, 10].

A dataset with strong phylogenetic signal is prerequisite for analyses of the evolution of taxa through time. So far, molecular clock analyses have delivered conflicting results concerning the origin and radiation ages of Squaliform sharks in general and the rise of families in particular [23, 24]. Molecular clocks are best calibrated using information from fossils or from vicariant biogeographic events. Squaliformes are well documented in the fossil record for sharks, which is largely comprised of teeth. Most Squaliform sharks display diagnostic clade specific dentitions pointing to high levels of trophic specialization and conservatism. A number of fossils can therefore be readily assigned to extant lineages such as the Gulper shark genus *Centrophorus* [25] or the Viper dogfish *Trigonognathus* [26], without the need to erect distinct genera for extinct forms whose phylogenetic affinities are unclear. According to [25], the fossil record of Squalidae extends back to the Upper Jurassic, while families Centrophoridae, Etmopteridae, Somniosidae, Oxynotidae, and Dalatiidae appeared rather instantaneously at the beginning of the Upper Cretaceous, which has been suggested to be a period of adaptive evolution in response to new ecological opportunities [23, 24]. The oldest Echinorhinid fossils are recorded from the Lower Cretaceous [25, 27] the evolution of bioluminescence in Kitefin (Dalatiidae) and Lantern sharks (Etmopteridae) appears to be correlated with the diversification of Squaliform sharks in the deep-sea [23, 24, 28, 29]. Surprisingly, it has not been clear at which point in their evolutionary trajectory, squaliform sharks first acquired photophores. Despite the fact that Shirai [8] had noted that all squaloid sharks except *Echinorhinus*, *Centrophorus*, *Cirrhigaleus*, *Deania*, *Somniosus*, and *Squalus* bear luminous organs, several recent studies suggested that photophores are only present in Etmopteridae and Dalatiidae [2, 23, 30, 31].

In this study, we estimate the phylogenetic interrelationships of Squaliform sharks by applying a gene capture approach that targets a large number of single-copy nuclear exons [32] to a generic level sampling of “squaloid” sharks and closely related taxa [8]. We have used these data in conjunction with fossil calibration data, to estimate times of divergence and diversification rates among the extant lineages examined. We have also explored the potential role that bioluminescence may have had in promoting diversification in these animals, by reconstructing ancestral character states based on the inferred tree and the presence of photophores in extant forms.

## Results and discussion

### Molecular phylogeny of Squaliformes

On average, 200,000 of 352,605 possible basepairs, were sequenced per specimen (Additional file 1: Table S1). Characteristics of the raw dataset are given in Additional file 1: Table S2. Missing data were randomly distributed among specimens resulting in a large amount of incomplete sequences per captured locus and specimen.

MARE [33, 34] detected 174 phylogenetically informative loci in the raw dataset (Additional file 1: Figure S1). Re-blasting the full genome of *C. milii* against the 174 phylogenetically informative loci resulted in two potentially paraloguous loci (cds 1200 (unknown) and cds 1366 (LRP4)). Excluding these two loci and repeating the maximum likelihood analysis as described above did not affect the inferred tree topology.

**Fig. 1:**
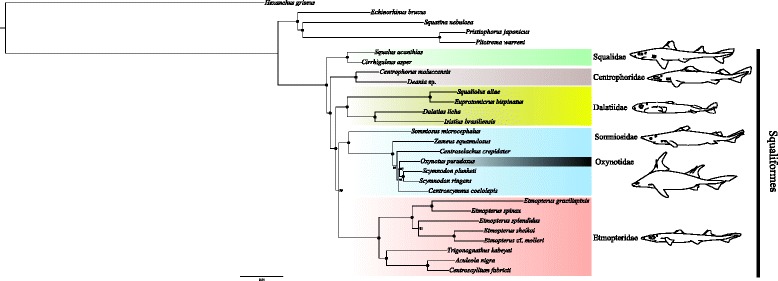
Maximum likelihood phylogenetic estimate of squalomorph sharks based on gene capture data of 172 nucleotide loci under a GTR + Gamma model using RAxML [35] partitioned into two sets, 1st and 2nd codon position as well as 3rd codon only. Analyzed specimens are listed in Additional file 1: Table S1. Nodes marked with black dots indicate 100 % bootstrap support and a posterior probability of 1 assessed in the Bayesian inference from the Phylobayes 3 analysis applying the CAT model [36, 37, 64]. Tree rooted midpoint, no outgroup defined.

Phylogenetic estimates presented herein provide a fully resolved and well-supported molecular hypothesis for the phylogeny of Squaliform sharks. The Maximum Likelihood trees as well as the Bayesian inferences resulting from different types of analyses carried out using RaxML [35] and PhyloBayes 3.3f [36, 37] were broadly congruent in topology except for the phylogenetic placement of *Oxynotus*. This taxon appears as sister taxon to all somniosid genera except for *Somniosus* in an analysis of all 1265 loci, but is nested among somniosid genera except for *Somniosus* in the analyses of the reduced dataset comprising 174 and 172 loci, respectively. The topology used for further analysis is summarized in Fig. 1, and is based on the 172 concatenated nucleotide loci that were selected through the MARE matrix reduction process and re-blasting analysis. The concatenated and aligned 172 nucleotide loci are deposited in the Dryad data repository [38] (Additional file 1: Tables S3 and S4, Figures S3 to S6).

This phylogenetic estimate reveals two major clades: the Squaliformes excluding Echinorhinidae and a clade containing *Squatina*, Pristiophoriformes, and *Echinorhinus* (Fig. 1). Within this clade, *Echinorhinus* is sister to *Squatina* and Pristiophoriformes. Results suggest that Echinorhinidae are not Squaliform sharks, but are the sister group to Angel- (Squatiniformes) and Saw sharks (Pristiophoriformes), as previously suggested by the analysis of mitochondrial data [21]. Therefore, Squaliformes form a monophyletic group only, if *Echinorhinus* is excluded. This study does not support results from [24], suggesting *Echinorhinus* being the sistergroup to the remaining Squaliform lineages. The node time estimation for the *Echinorhinus* lineage suggests an Upper Jurassic splitting of the extant *Echinorhinus* lineage and the *Squatina* plus Pristiophoriformes clade. This dates the *Echinorhinus* lineage older than anticipated from the fossil record, which reports the oldest echinorhinid fossil from the early Cretaceous (Hauterivian) of southeastern France [27], while the oldest squatinids already appear in the Upper Jurassic [25].

Within the Squaliform clade, the first split separates Squalidae from the remaining families Centrophoridae, Etmopteridae, Dalatiidae, Somniosidae, and Oxynotidae. The genera *Squalus* and *Cirrhigaleus* appear as sister taxa. Centrophoridae split from Etmopteridae, Dalatiidae, Somniosidae, and Oxynotidae, where genera *Deania* and *Centrophorus* are sister. Dalatiidae are sister to a clade comprising Etmopteridae, Somniosidae, and Oxynotidae. There are two clades within the dalatiids, one comprising the *Isistius* and *Dalatias* lineages, the other *Squaliolus* and *Euprotomicrus*. As shown in Fig. 1, Somniosidae *sensu stricto* form two clearly distinct lineages that are sister to each other, one containing the genus *Somniosus* (Fig. 1), the other lineage contains all other remaining somniosid genera. Oxynotidae cluster within Somniosidae (Fig. 1). Within Etmopteridae, *Trigonognathus* is sister to a clade comprising *Aculeola* and *Centroscyllium. Etmopterus* is sister to this previously described clade forming four distinct lineages representing the subclades described in [23].

*Oxynotus* is inferred to be nested within Somniosidae, rendering the family Somniosidae paraphyletic (Fig. 1) in the current study. This result is repeatedly recovered in phylogenetic estimates based on DNA sequence data (both mitochondrial and nuclear) [19–24]. Given the consistency of the inferences from molecular data, it would be interesting to see if any anatomical features also support the link between Oxynotidae and Somniosidae. *Oxynotus* clusters with a group of otherwise morphologically similar species of somniosids, i.e. along with *Zameus*, *Centroselachus*, *Scymnodon*, and *Centroscymnus*. Our molecular results show that all five genera are closely related (Fig. 1). This is especially evident when comparing intergeneric diversity within Somniosidae with the large intrageneric sequence differences evident within the genus *Etmopterus* (Fig. 1). Moreover, there are limited morphological characters that can be used to differentiate some of these taxa [8, 39]. Together these results imply that assigning separate generic status to some species within Somniosidae may be an overrepresentation of the true diversity within the family.

### Occurrence and significance of bioluminescence in Squaliform sharks

The Bayesian inference estimated with BEAST [40, 41] is widely congruent with the maximum likelihood phylogeny (Fig. 1, Additional file 1: Figures S3 to S6).

Results from node time estimates based on 172 loci support a squaliform shark radiation beginning in the Lower Cretaceous and continuing through to the Upper Cretaceous (Table 1). A sister-group relationship of non-luminous Squalidae with a clade comprising all other Squaliformes is strongly supported as the most ancient split of extant Squaliformes (Table 1) and is consistent with the fossil record [25, 29]. Centrophoridae rise in the Lower Cretaceous, followed by the splitting of Dalatiidae, Somniosidae, Oxynotidae and Etmopteridae, which also aligns with the sequence of appearance of these taxa in the fossil record. However, 95 % confidence intervals are large, preventing exact estimates (Table 1). A second radiation occurred within Etmopteridae and Somniosidae in the Upper Cretaceous and the beginning of the Palaeocene (Table 1), again, a time period characterized by profound changes in the marine environment including the deep-sea. As discussed in [23], the Eocene recovery phase and the admixing of the deep-sea by the establishment of the circum-antarctic current at the beginning of the Oligocene, may have set the stage for this second radiation.

**Table 1:** Node time estimates for major splitting events

Nr.	Node	Node age	95 % HPD	Series/Epoch
1	Squalomorphii	202.8	190 – 241.32	Middle Triassic to Lower Jurassic
2	Splitting of Squaliformes from the clade comprising *Echinorhinus, Squatina, Pliotrema & Pristiophorus*	177.34	153.85 – 203.99	Upper Triassic to Upper Jurassic
3	Clade comprising *Echinorhinus, Squatina, Pliotrema & Pristiophorus*	147.59	145-156.1	Upper Jurassic
4	Radiation Squaliformes	132.86	130 – 143.18	Lower Cretaceous
5	Split Centrophoridae from Dalatiidae, Etmopteridae, Oxynotidae & Somniosidae	126.68	113.94 – 137.88	Lower Cretaceous
6	Split Dalatiidae from Etmopteridae, Oxynotidae & Somniosidae	116.1	99.2 – 131.01	Transition Lower to Upper Cretaceous
7	Split Etmopteridae from Somniosidae & Oxynotidae	110.51	92.81 – 124.88	Transition Lower to Upper Cretaceous
8	Split *Centrophorus* from *Deania*	90.82	89 – 96.84	Upper Cretaceous
9	Split *Somniosus* from Oxynotidae & remaining Somniosidae	92.29	64.8 – 114.49	Upper Cretaceous
10	Radiation Dalatiidae	83.57	65 – 105.4	Upper Cretaceous
11	Radiation Etmopteridae	77.15	65 – 90.66	Upper Cretaceous
12	Radiation *Etmopterus*	60.38	46.28 – 74.64	Upper Cretaceous to Palaeocene
13	Split *Trigonognathus* from *Aculeola & Centroscyllium*	61.5	44.5 – 76.86	Upper Cretaceous to Palaeocene
14	Radiation Somniosidae excluding *Somniosus*	43.4	24.46 – 63.94	Eocene
15	Split *Oxynotus* from *Scymnodon*	28.91	15.32 – 47.11	Oligocene

**Fig. 2:**
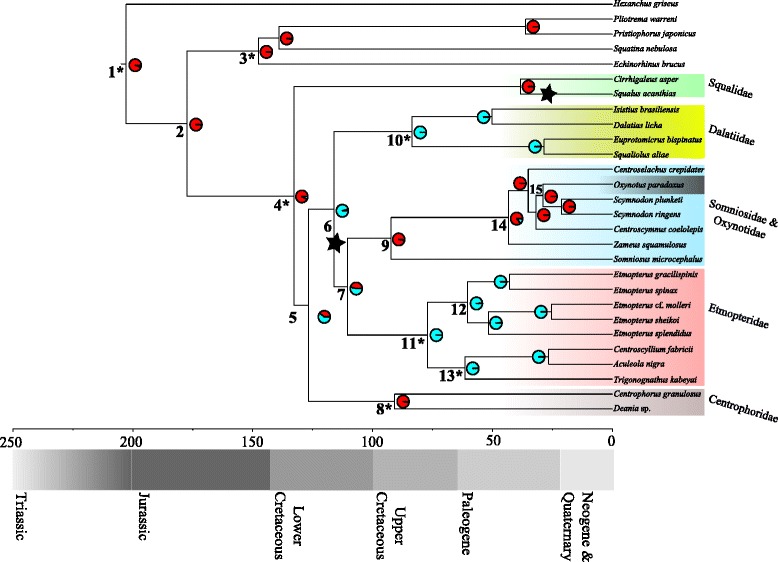
Chronogram resulting from the BEAST [38] analysis with estimated shift in the diversification rate. Background rate *r* = 0.02. The black stars indicate significant increase in the diversification rate to *r* = 0.15 (radiation Squalidae) and *r* = 0.05 (Etmopteridae, Oxynotidae and Somniosidae) estimated with MEDUSA [39, 40]. Scale bar in millions of years. Numbers at branches refer to node numbers given in Table 1. Pie charts indicate the probability that ancestral taxa are luminescent (blue) or not (red). Families Etmopteridae and Dalatiidae were coded as luminous as well as the genus *Zameus* within Somniosidae. * = Node calibrated with information from the fossil record (Table 2)

Novel ecological opportunities after oceanic anoxic events have been hypothesized to trigger adaptive radiation of sharks in deep-water environments in the Lower Cretaceous [10, 24]. Results from the MEDUSA [42, 43] analysis indicate a background diversification rate *r* = 0.02. An elevated diversification rate was detected for families Etmopteridae, Dalatiidae, Oxynotidae and Somniosidae, (*r* = 0.05) and the radiation of the species-rich genus *Squalus* (*r* = 0.15, Fig. 2).

We reconstructed ancestral character states in order to test the hypothesis that bioluminescence evolved in conjunction with the diversification of the Dalatiidae, Etmopteridae, Oxynotidae and Somniosidae. In the first analysis, we coded Dalatiidae and Etmopteridae as luminescent. Results from this analysis indicated that the common ancestor of families Dalatiidae, Etmopteridae, Oxynotidae, and Somniosidae was already likely carrying luminous organs. Interestingly, Somniosidae have been widely accepted as non-luminous [2, 23, 30, 31, 44]. However, Shirai [8] suggested that all Somniosidae are luminescent except for the genus *Somniosus*, which may have secondarily lost the ability to produce light.

**Fig. 3:**
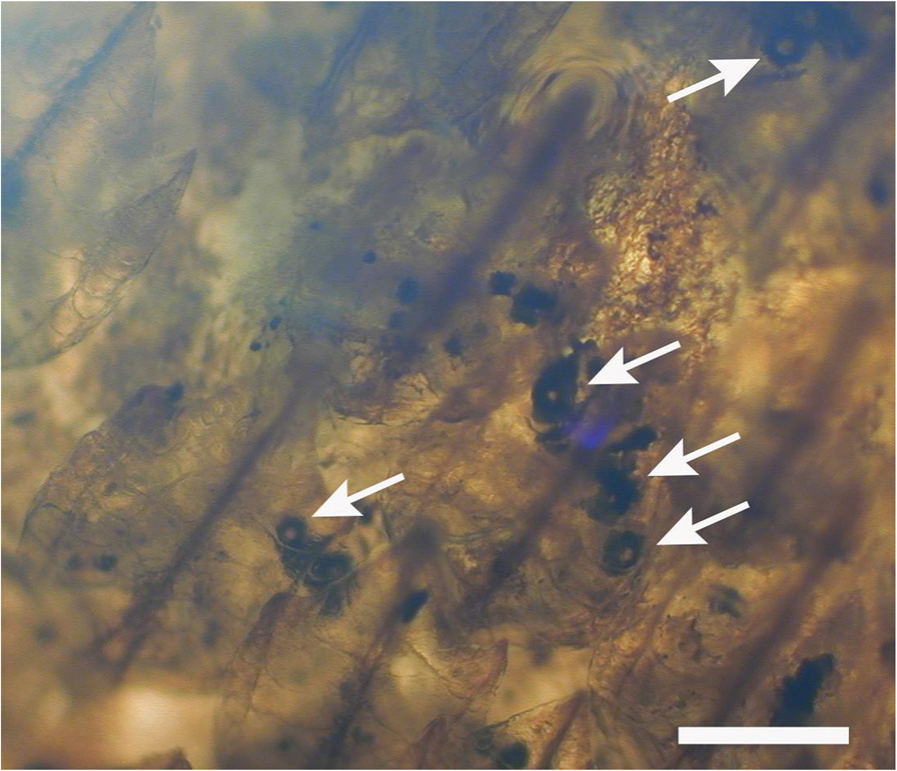
Microscopic photograph of an excised ventral skin patch of *Zameus squamulosus* (ZSM30966). Arrows indicate photophores in open state. Scale bar indicates 200 μm

We reviewed the presence of photophores in Somniosidae and Oxynotidae, by inspecting the ventral surface area of several specimens housed in zoological collections. The inspection of skin samples from *Zameus squamulosus* revealed clear presence of epidermal photophores (mean diameter = 41.75 ± 1.95 μm, density = 26 units mm^−2^, PAP = 3.74 %) in this taxon (Fig. 3). The majority of these organs appeared to be ring-shaped and covered with translucent dermal denticles. *Zameus* photophores are visible as open dark circular plaques, typical of functional photophores that are capable of producing light. Indeed, this morphology is typically adopted by dalatiid and etmopterid photophores while glowing [44–48]; the translucent nature of *Z. squamulosus* scales would allow efficient transmission of underlying photophore light, similar to the observation of light transmission through the ventral scales of opisthoproctid fishes [49] or through the dorsal finspines of the velvet belly lanternshark, *Etmopterus spinax* [50].

Morphological data presented herein provide clear evidence that functional photophores are present within Somniosidae, at least within the genus *Zameus* (Fig. 3). All other inspected specimens showed no evidence of epidermal photophores. In light of this, the ancestral character state reconstruction was repeated incorporating results from the inspection of skin samples, i.e. coding the genus *Zameus* in addition to Etmopteridae and Dalatiidae as luminescent. Results from this analysis further increased the likelihood that the common ancestor of Dalatiidae, Etmopteridae and Somniosidae was luminescent (Fig. 2). The common ancestor of Centrophoridae, Etmopteridae, Dalatiidae, Oxynotidae, and Somniosidae is also implied to have been luminescent, but the likelihood is less compelling. A further analysis following [8] coding somniosid genera *Centroselachus*, *Centroscymnus*, *Scymnodon*, and *Zameus* as luminous further increases the likelihood so that the common ancestor of all Squaliformes except Squalidae may already have been luminescent (Additional file 1: Figure S8). This indicates that extant Centrophoridae may have secondarily lost their ability to emit light, i.e. that luminous organs may have already been present at the branching event giving rise to families Centrophoridae, Dalatiidae, Etmopteridae, Somniosidae, and Oxynotidae (Fig. 2). This suggests that luminescence evolved along and facilitated the Squaliform deep-sea radiation – a scenario that would be consistent with the elevated diversification rate detected for Etmopteridae, Somniosidae, and Oxynotidae. (Fig. 2, Additional file 1: Figures S8 and S9). We speculate that the common ancestor of families Dalatiidae, Etmopteridae, Oxynotidae, and Somniosidae was luminescent and used this to enhance camouflage by counterillumination as this is assumed to be the most basal function of shark bioluminescence [23, 28, 45, 47].

The occurrence of bioluminescence within the family Somniosidae is not surprising as especially the smaller sized genera (*Centroselachus*, *Centroscymnus*, *Scymnodon,* and *Zameus*) occur in sympatry with other luminous sharks such as etmopterids and dalatiids as well as a number of other luminescent deep-sea taxa including myctophid fishes which interestingly were estimated to have radiated in a similar time window [51]. Results presented here lend further support to the hypothesis that bioluminescence in sharks evolved only once [29, 47]. Work in progress will allow identifying all luminous taxa within the family Somniosidae.

## Conclusions

Our findings provide insights into the phylogeny of Squaliform sharks as well as the evolution of bioluminescence in the group. The radiation is estimated to have started in the Lower Cretaceous and continued through to the Upper Cretaceous. The initial elevated diversification rate is correlated with the likely first occurrence of luminous organs in sharks. The presence of photophores was confirmed for the genus *Zameus* in the family Somniosidae, implying that bioluminescence in sharks is not restricted to families Etmopteridae and Dalatiidae as is widely believed.

## Methods

### Targeted gene capturing

To ensure correct sample IDs of target samples, we either used genomic DNA of specimens which were previously analyzed in [21, 23, 52, 53] or generated NADH2 sequences as described in [22] and compared to the samples analysed in [21, 52, 53]. In the latter case, genomic DNA was extracted from collection material (tissues in GJPN tissue collection) already used in previous studies and stored in 95 % ethanol. Genomic DNA was obtained using the Promega Wizard ® DNA Purification System (Fisher Scientific). Total amounts of DNA were measured using a Qbit® Fluorometer (Life Technologies).

Subsequently, genomic DNA of the 28 target samples was sheared to approximately 500 bp using a Covaris® Sonicator. Sheared samples were used to prepare Illumina sequencing libraries following the protocol provided in [32]. See Additional file 1: Table S1 for an overview of samples analysed.

We designed custom RNA bait libraries for targeting putatively single-copy orthologous genes based on sequences derived from seven shark species in [32], i.e. *Chlamydoselachus anguineus*, *Etmopterus joungi*, *Isurus oxyrinchus*, *Orectolobus halei*, *Carcharhinus amblyrhynchos*, *Heterodontus portusjacksoni*, and *Squatina nebulosa*. Each bait library comprised a pooled series of 120 bp baits designed for each target locus. As in [32], a 60 bp tiled overlap across baits was used to generate two-fold redundancy coverage for each target gene. When the length of the target gene was less than 120 bp, the sequence was extended in length to 120 bp by adding thymine nucleotides. The baits were manufactured by MyCroarray® (Ann Arbor, MI, USA).

Thereafter, gene capture was conducted by hybridization of target DNA to the baits. After hybridization, unbound and non-target DNA was washed away [32]. The remaining library was enriched for target loci and was re-amplified to incorporate sample specific indices. Samples were pooled in equimolar ratios for sequencing. The pooled product was quantified using the CFX Connect Real-Time PCR system (Bio-Rad, Hercules, CA). Pooled sample was diluted to 8 pM and used for paired-end 150 bp or 250 bp sequencing on an Illumina MiSeq sequencing instrument (Illumina, Inc, San Diego, CA). Sequence reads associated with each sample were identified by their respective indices.

### Alignment reconstruction of gene capture data

Adapters were trimmed from sequence reads using Trimgalore v0.3.7. [54, 55] and assembled de novo using ABySS ver1.3.5. [56] with a k-mer of 64. Assembled contigs were assigned to core ortholog groups using HaMStR [57]. The core ortholog database consisted of profile hidden Markov models of orthologous sequence groups from model vertebrates [32]. Any sequence that matched a core-ortholog pHMM was provisionally assigned to the corresponding orthologous group. In order to be retained in the final matrix, provisional sequence hits also had to satisfy a reciprocal best BLAST criterion when compared to *Callorhinchus milii* as the reference taxon. Orthologous exons were trimmed from non-target intron information and aligned with Mafft [58, 59]. Finally, all loci were concatenated.

### Data analysis and phylogenetic reconstruction

Maximum likelihood (ML) trees were estimated using RAxML GUI [33, 60]. The initial ML analysis used the complete concatenated dataset (i.e. 1265 loci and 28 taxa) under GTR GAMMA using different partitioning schemes (Additional file 1: Table S4) using the automatic halt for bootstrapping [61]. Squalomorph sharks are widely accepted as monophyletic [8, 19–22]. Within Squalomorphs, Hexanchiformes are considered to form the most basal lineage [8, 20–22], therefore, *Hexanchus griseus* was chosen as the outgroup taxon.

Subsequently, MARE ver.1.2 [33, 34] was used to examine the dataset for phylogenetically informative sites and taxa. MARE [33, 34] was designed to identify the most phylogenetically informative subset of sites contained in phylogenomic data sets. It is especially well-suited to analysis of data sets with a high proportion of missing data. MARE [33, 34] identified 174 maximally informative loci for our data set reducing the maximal total sequence length per specimen from 352,605 bp to 73,925 bp (Additional file 1: Figure S1).

As a further scan for paraloguous sequences, we re-blasted the full genome of *Callorhinchus milii* against the remaining 174 loci to check, if each sequence has only a single hit in the *C. milii* genome using customized Perl scripts (Additional file 1).

The reduced nuclear dataset comprising 172 concatenated nucleotide loci is deposited at [38]. This data was analysed as described for the full dataset and additionally RaxML GUI [35, 60] was applied to the amino acid alignment comprising infomative loci using the best partitioning scheme suggested by PartitionFinder Protein v1.1.1 [62, 63]. Further, the reduced 172 loci DNA sequence data alignment was analysed with PartitionFinder v1.1.1 to look for best fitting partition schemes and models of molecular evolution [62, 63] to determine if different partition types influence the tree topology. We used the rcluster option with a rcluster percentage of 10 [63] for the analysis. The Bayesian mixture model CAT [64] implemented in PhyloBayes 3.3f [36, 37] was used on the concatenated 172 amino acid alignment to partition sites into different rate categories using non-parametric modeling of site specific effects. This allowed us a topological comparison to the ML analysis under GTR GAMMA [65]. Four independent chains were run in parallel. The tracefiles and treelists of all four chains were used to check for convergence . The analysis was stopped with a maximum difference of 0.16 and effective sample sizes exceeding 100, with the exception of the allocent statistic (see Additional file 1). A majority rule consensus tree was computed from 12997 input trees from each chain with a burn-in of 1000 trees and analyzing every second tree of the pooled trees. The consensus tree was rooted midpoint.

See Additional file 1: Table S4 and Figures S3 to S7 for a summary of partitioning schemes and phylogenetic analyses conducted. To ensure that the choice of a single outgroup does not have a negative effect such as long branch attraction on our phylogenetic analysis, we performed analysis not defining an outgroup, defining different outgroups as well as deleting *Hexanchus griseus* from the dataset and re-computing a phylogenetic estimate without defining an outgroup taxon (Additional file 1: Figure S2).

### Node time estimation and diversification rate

BEAST ver. 1.8.0 [40, 41] was used to estimate node ages from the MARE [33, 34] reduced nucleotide alignment excluding cds 1200 and 1366 and partitioned into two partitions, the first comprising codon positions 1 and 2, the second codon position 3 applying the GTR Gamma model. XML files were created in BEAUTi [41]. The analysis assumed a relaxed molecular clock approach under an uncorrelated lognormal model [40]. The Yule speciation process was implemented assuming a constant speciation rate per lineage as tree prior. We calibrated our phylogenetic tree using calibration points deployed from the fossil record of Squalomorph sharks (Table 2). The root age calibration should reflect the age of origin of Squalomorph sharks. The discussion of the age of Squalomorphs is enduring and contingent on the discovery of new fossil information [25]. Here, we assumed the origin of Squalomorphii, i.e. our root age, to have occurred between 190 and 279 Ma based on distinct Hexanchoid teeth from the Lower Jurassic as minimum age and the fossil appearance of *Protracodus*, the oldest tooth fossils that carry morphological characters of euselachians as a soft upper bound. We would like to point out that we consider this calibration as a minimum age calibration for the crown Squalomorphii following [25, 66] even though the chondrichthyan stem may be as old as, or even older than, the Middle Ordovician [67].

**Table 2:** Calibration points used for node time estimates of squaloid sharks

	Taxon set	Minimum age (Ma)	Soft upper bound (Ma)	Citation
Cp 1	Root age (Squalomorphii)	190	279	[71]
Cp 2	Squaliformes	130	163	[25]
Cp 3	Echinorhinidae, Squatinidae & Pristiophoridae	145	163	[25]
Cp 4	Centrophoridae	89	100	[72]
Cp 5	Dalatiidae	65	100	[25]
Cp 6	Etmopteridae	65	100	[25]
Cp 7	*Trigonognathus, Aculeola & Centroscyllium*	44.5	100	[26]

The clade comprising Echinorhinidae, Pristiophoriformes *Pliotrema* and *Pristiophorus* as well as *Squatina* was assumed to vary in age between 145 to 163 Ma (Upper Jurassic) based on articulated fossils of Squatinids at the lower and *Echinorhinus* sp. teeth at the upper end of the time frame [25, 27]. The minimum age of Squaliformes was calibrated to 130 Ma based on the fossil taxon *Protosqualus* with a soft upper bound at 163 Ma allowing for the possibility that Squaliform sharks were already present in the Upper Jurassic [25]. Squaliform family-level diversity is assumed to have originated in the late Mesozoic (Upper Cretaceous) [23], while most extant genera likely originated in the Cenozoic. Fossil evidence was used to calibrate the minimum age of Centrophoridae, Etmopteridae and Dalatiidae to be 65 Ma (C/T boundary) with a soft upper bound of 100 Ma (beginning of the Upper Cretaceous). Further, the clade comprising *Trigonognathus*, *Aculeola*, and *Centroscyllium* was assumed to be of minimum age of 45 Ma and a lower bound of 100 Ma based on the fossil record of *Trigonognathus virginiae* [26] and the age estimate of extant Etmopteridae in [24].

All analyses assumed an exponential prior distribution for calibration points. Three independent runs were performed with a Markov Chain lasting 90 million generations each, sampling trees every 1000 generations. One run included the maximum likelihood inferred tree with the highest likelihood as a newick formatted starting tree. This BEAST input file is deposited in the Dryad data repository [36]. Combined log files were analyzed in Tracer v.1.6 [68] to check, if the effective sample sizes (ESS) of parameters represent the posterior distribution adequately; further trace and density plots were checked for convergence of the MCMC and posterior probability distributions in different runs. After defining a burn-in of 25 % of all sampled trees in each run, TreeAnnotator [40] was used to create a consensus tree which was visualized in FigTree v.1.4.0 [69].

We used the R [70] module MEDUSA (modeling evolutionary diversification using stepwise AIC) [43] implemented in the GEIGER package [42] to estimate changes in the diversification rate based on the consensus chronogram attained from the BEAST [41] analysis. Species richness values were obtained from [1].

### Ancestral bioluminescence within Squaliformes

Ancestral character states of bioluminescence were reconstructed using Maximum Likelihood estimates implemented in the R [70] package GEIGER [42] and are based on the chronogram attained from the BEAST [41] analyses. In a first analysis, we coded only Dalatiidae and Etmopteridae as luminescent. Results from this analysis indicated that the common ancestor of families Dalatiidae, Etmopteridae, and Somniosidae was already likely luminescent. As an empirical test of this idea, we subsequently inspected the ventral surface area of Somniosidae and Oxynotidae specimens from the Bavarian State Collection of Zoology –*Centroselachus crepidater* (ZSM30842), *Centroscymnus owstonii* (ZSM36725), *Oxynotus bruniensis* (ZSM30862), and *Zameus squamulosus* (ZSM30966)– and the Zoological Museum Hamburg –*Somniosus microcephalus* (ZMH 123507), *S. rostratus* (ZMH 25751), *Centroscymnus coelolepis* (ZMH 119748), *Centroscymnus owstonii* (ZMH 104894), *Centroselachus crepidater* (ZMH 103185), *Scymnodalatias* sp. (ZMH 122774), *Zameus squamulosus* (ZMH 120262; ZMH 120485). When pigmentation was apparent, a 1 cm^2^ skin patch was excised from the ventral surface of the specimen and observed under a binocular microscope (Leica MZ6, Wetzlar, Germany). If photophores were observed, a picture was taken and analysed in Image J v. 1.46 using a random 1 × 1 mm counting frame to estimate photophore mean diameter, photophore density and proportion of the skin surface area occupied by photophores (PAP) following the method of [28]. Thereafter, the ancestral character state reconstruction was repeated incorporating results from the inspected skin samples in a second analysis, and incorporating information on presence of luminous organs in somniosids following [8] in a third test. See Additional file 1 for documentation on R scripts used and the different photophore presence/ absence matrix (Additional file 1: Table S6).

## Availability of supporting data

The data sets supporting the results of this article are available in the Dryad repository, http://datadryad.org/review?doi=doi:10.5061/dryad.n3581. See also Additional file 1.

## Additional file

Additional file 1:
**Documentation of conducted analyses.** (PDF 1710 kb)
