# Supplementary material for: Molecular phylogeny of Squaliformes and first occurrence of bioluminescence in sharks
Source: BMC Evol Biol. 2015 Aug 16;15:162. doi: 10.1186/s12862-015-0446-6 (PMC4537554; doi:10.1186/s12862-015-0446-6)
Supplement: Additional file 1: — Documentation of conducted analyses. (PDF 1710 kb) [file 12862_2015_446_MOESM1_ESM.pdf]

Additional file 1: analyses documentation

Table S1. List of species analysed.

Genus	Species	GJPN tissue collection number
Squalus	*acanthias*	GN6336
Cirrhigaleus	*asper*	GN3831
Centrophorus	*moluccensis*	GN8086
Deania	sp.	GN7456
Dalatias	*licha*	GN1006
Isistius	*brasiliensis*	GN3748
Squaliolus	*aliae*	GN6176
Euprotomicrus	*bispinatus*	GN3749
Somniosus	*microcephalus*	GN1156
Zameus	*squamulosus*	GN7131
Scymnodon	*ringens*	GN1703
Centroselachus	*crepidater*	GN7127
Centroscymnus	*coelolepis*	GN6626
Scymnodon	*plunketi*	GN5607
Oxynotus	*paradoxus*	GN1852
Aculeola	*nigra*	GN7450
Centroscyllium	*fabricii*	GN8195
Etmopterus	*splendidus*	GN10139
Etmopterus	*sheikoi*	GN12278
Etmopterus	cf. *molleri*	GN10014
Etmopterus	*spinax*	GN5162
Etmopterus	*gracilispinis*	GN7458
Trigonognathus	*kabeyai*	GN12280
Echinorhinus	*brucus*	GN1983
Pliotrema	*warreni*	GN7338
Pristiophorus	*japonicus*	GN1049
Squatina	*nebulosa*	GN10161
Hexanchus	*griseus*	GN2342

Table S2. Completeness statistics for captured data, raw dataset (cds = coding sequences; bp = basepairs).

**Average number of genes captured:**	1000	Out of 1265 possible
**Completeness by cds:**	74.57%	27,357 cds present out of 36,685
**Completeness by position in alignment:**	59.59 %	6,163,401 positions present out of 10,343,865
**Average sequence length per specimen:**	201,099 bp	Out of 352,605 possible

Search for phylogenetically informative loci using MARE

```
MARE OUTPUT
original multiple sequence alignment
-----------------------------------
information content of B     : 0.11
matrix saturation            : 0.746
nr of taxa                   : 29
nr of partitions             : 1264

Parameters
----------
weighting of inform. content : 3
weighting of taxa            : 1
number of evaluated quartets : 20000

reduced multiple sequence alignment
----------------------------------
information content of B'            : 0.357
nr of taxa                           : 29
nr of partitions                     : 174
ratio of reduced and complete matrix : 0.138
matrix saturation                    : 0.922
```

Figure S1: Overview of the MARE [33, 34] analysis. Rows show analysed taxa, columns different loci. Each locus is represented for each taxon by a cell. Cell colors code for the information content of loci: white cells = gene absent; red cells = treelikeness equals 0; different stages of blue to black cells = treelikeness is 0.1 – 1.

(A) Information content (treelikeness) for the full set of 1265 loci and 29 taxa.

(B) Reduced dataset after exclusion of uninformative loci (174 loci).

For a high resolution image please download Figure S1 directly from the Dryad data repository: http://datadryad.org/review?doi=doi:10.5061/dryad.n3581

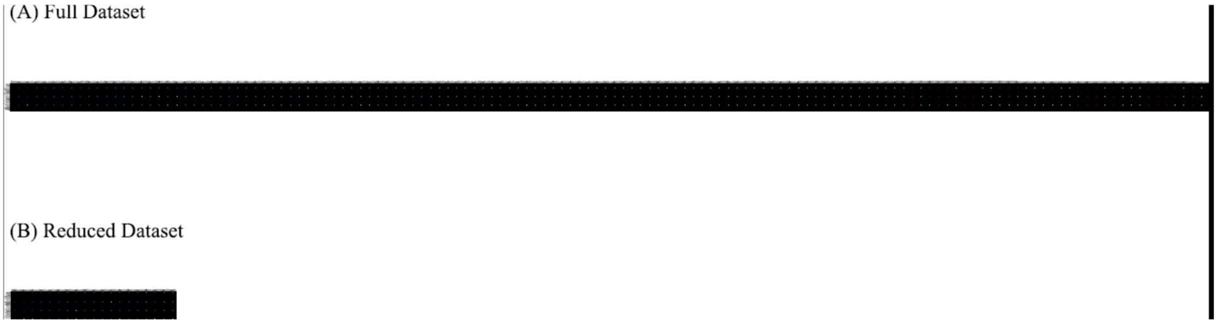

PERL Script used to re-blast 174 loci resulting from the MARE analysis against the reference genome of *Callorhinchus milii* to check for multiple hits

```
#!/usr/bin/perl -w
#
# Name: reblast.pl
#
# This script is used to re-blast blastout sequences of each gene to the genome of designing baits and bin sequences into genes.
#
# Datafile: fasta file of the query and fasta files of the genes
#
# Input: the subject name and query name
#
# Output: blastn output file in format 6, sequences binned as genes.
#
# sample: ./reblast.pl -subject="Oreochromis_niloticus" -query="Gobioidei"
#
# Written by
#           Chenhong Li, Ting Kuang
#           Shanghai Ocean University, China.
#           Created on Sep 2014
#           Last modified on
use strict;
use warnings;
use Getopt::Long;   # include the module for input
```

```
my $subject; #genome sequence need make blast database
my $query; #folder containing all gene data file
my $opt = GetOptions( 'subject:s', \$subject,
                      'query:s', \$query); #set command line options

my $resultdir = $query . ".result";
`mkdir ../genebin/$resultdir`;#make dir for the output
my $resultlog = $query . ".log";
`mkdir ../genebin/$resultlog`;#make dir for the para log


#call outside program makeblastdb
#`makeblastdb -in ../subject/$subject.fas -out ../blastdb/$subject -dbtype nucl`;

my $que = "../query/$query";
opendir (DIR, $que) or die $!; #open folder containing all gene data file

while (my $file = readdir (DIR)) { #read all gene file under local alignment out folder
   next if ($file =~ /^\./); #skip files beginning with
   (my $gene) = $file =~ /(\S+)\.fas/;
   my @file = split (/\s/, $file); #split the names of gene
   chomp(@file);
   my @sortedfile = sort @file;


   foreach my $file (@sortedfile) {##call outside program blastn

      `blastn -query $que/$file -db ../blastdb/$subject -out ../blastout/$gene.blast.out.txt -word_size 7 -
gapopen 5 -gapextend 2 -penalty -1 -reward 1 -evalue 0.000001 -outfmt 6`;


      my ($BLAST_FILE, $FASTA_FILE, $RESULT_FILE, $RESULT_LOG);
      my $blastout = "../blastout";
```

```
my $blast = "$blastout/$gene.blast.out.txt";


#open 4 file: $new, $blast, $path and $log
my $new = "../genebin/$resultdir/$gene.fas";
open $RESULT_FILE, ">$new" or die ("Cannot open $new for writing ($!)");
open $BLAST_FILE, "<$blast" or die ("Cannot open $blast for reading ($!)");
my $log ="../genebin/$resultlog/$gene.fas";
open $RESULT_LOG, ">$log" or die ("Cannot open $log for writing ($!)");
my $path = "../query/$query/$file";
open $FASTA_FILE, "<$path" or die ("Cannot open $path for reading ($!)");


my $line = readline ($FASTA_FILE);#read line ">query$subject"
chomp $line;
print $RESULT_FILE "$line\n";#store line ">query$subject" to $new
my $seqline = readline ($FASTA_FILE);#read sequence line of query$query
chomp $seqline;
print $RESULT_FILE "$seqline\n";##store sequence line of query$query to $new
my %note_seq;
while (my $newline = readline ($FASTA_FILE)) {
    chomp $newline;
    if ((my $speciesname) = $newline =~ /^>(\S+)/) {
        my $newseqline = readline ($FASTA_FILE);
        chomp $newseqline;
        $note_seq{$speciesname} = $newseqline;
    }
}



my $bline = readline ($BLAST_FILE);#read the first line "query$query" in file $blast
chomp $bline;
my ($species, $seqid, $pos1, $pos2) = $bline =~
/(\S+)\s+(\S+)\s+\S+\s+\S+\s+\S+\s+\S+\s+\S+\s+\S+\s+(\S+)\s+(\S+)\s+\S+\s+\S+/;
($pos1, $pos2) = ($pos2, $pos1) if ($pos1 > $pos2);
```

```
        my $nspecieslag = "a";
        while (my $newline = readline ($BLAST_FILE)) {
            chomp $newline;
            my ($nspecies, $nseqid, $npos1, $npos2) = $newline =~
/(\S+)\s+(\S+)\s+\S+\s+\S+\s+\S+\s+\S+\s+\S+\s+\S+\s+(\S+)\s+(\S+)\s+\S+\s+\S+/;
            ($npos1, $npos2) = ($npos2, $npos1) if ($npos1 > $npos2);

            next if ($nspecies eq $nspecieslag);
            if ($nseqid ne $seqid) {
                print $RESULT_LOG ">$nspecies\n$note_seq{$nspecies}\n";
            } else {
                if ($npos2 <= $pos1) {
                    print $RESULT_LOG ">$nspecies\n$note_seq{$nspecies}\n";
                } elsif ($npos1 >= $pos2){
                    print $RESULT_LOG ">$nspecies\n$note_seq{$nspecies}\n";
                } else {
                    print $RESULT_FILE ">$nspecies\n$note_seq{$nspecies}\n";
                }
            }
            $nspecieslag = $nspecies;
        }

        close ($BLAST_FILE) or die "Can't close $blast";
        close ($FASTA_FILE) or die "Can't close $path";
        close ($RESULT_FILE) or die "Can't close $new";
        close ($RESULT_LOG) or die "Cant't close $log";

    }
}

closedir (DIR);
```

Information on single loci used for phylogenetic and all subsequent analyses

Table S3A: Information on single alignments of 172 loci used for final analyses:

	# CDS	External name	# Sequences in alignment	Query Sequence Length (bp)	Max Sequence Length captured (bp)	Min Sequence Length captured (bp)
**1**	cd23	CDH23	28	453	450	111
**2**	cd28	UNC13C	28	1428	1413	495
**3**	cd30	PLCE1	28	681	681	243
**4**	cd55	DNAH1	25	189	189	99
**5**	cd66	SLC35B2	24	648	606	36
**6**	cd99	MED7	28	519	516	123
**7**	cd118	ALG11	27	252	252	117
**8**	cd120	MYCBP2	24	222	222	141
**9**	cd127	DYNC1H1	27	210	210	126
**10**	cd137	ABC1	20	213	213	93
**11**	cd153	CHTF18	21	210	207	123
**12**	cd170	ASB2	27	483	480	123
**13**	cd191	DNAH10	26	231	231	120
**14**	cd203	GUCD1	24	228	228	132
**15**	cd236	SMCR8	27	423	423	123
**16**	cd258	INTS2	26	222	222	90
**17**	cd267	ABCC3	26	300	300	117
**18**	cd272	CEP192	20	204	201	105
**19**	cd284	ACOX1	18	204	204	96
**20**	cd285	FLI1	25	204	201	93
**21**	cd295	BAIAP2	23	225	225	72
**22**	cd299	ZZEF1	15	258	258	168
**23**	cd300	DHX33	24	258	255	165
**24**	cd302	SUPT5H	25	198	198	111
**25**	cd324	AP4B1	25	226	223	1
**26**	cd329	RP2	28	474	471	123
**27**	cd330	TMEM60	26	378	375	69
**28**	cd334	TGFBRAP1	28	678	678	159
**29**	cd353	DYNC2H1	27	375	375	171
**30**	cd354	CTSC	27	591	498	186
**31**	cd357	DSTYK	27	375	375	135
**32**	cd362	EPRS	20	207	207	132
**33**	cd388	AMDHD1	28	276	273	129
**34**	cd393	MIEF1	26	219	216	69
**35**	cd404	ZDHHC23	26	204	204	120
**36**	cd405	IMPG2	26	207	207	93
**37**	cd426	VWA1	27	531	495	207
**38**	cd438	PANX2	28	1203	1200	396

	# CDS	External name	# Sequences in alignment	Query Sequence Length (bp)	Max Sequence Length captured (bp)	Min Sequence Length captured (bp)
**39**	cd441	VPS13D	28	696	693	120
**40**	cd447	PEX2	28	831	828	141
**41**	cd451	NAGLU	28	561	561	201
**42**	cd465	MON2	28	621	618	183
**43**	cd469	TMEM168	28	504	504	129
**44**	cd472	RAG1	28	1650	1650	168
**45**	cd473	YOD1	28	606	606	147
**46**	cd481	LRRC30	27	717	714	201
**47**	cd484	TGFBRAP1	27	336	336	162
**48**	cd485	NPC1	28	219	219	114
**49**	cd487	SS18	25	219	216	42
**50**	cd490	PRKDC	27	198	198	108
**51**	cd494	CYP7A1	28	546	546	162
**52**	cd506	BAMBI	26	285	282	147
**53**	cd514	TTN	27	270	270	144
**54**	cd520	MALRD1	24	231	231	69
**55**	cd521	C10orf112	27	366	363	120
**56**	cd522	OSGEPL1	28	381	381	126
**57**	cd550	POMGNT2	28	1728	1728	123
**58**	cd554	NPHP3	23	240	240	147
**59**	cd564	SCAP	27	319	319	97
**60**	cd567	MOCOS	27	654	642	123
**61**	cd568	EXOC3	28	750	675	147
**62**	cd570	ALG2	27	894	894	147
**63**	cd571	TDP2	21	237	237	147
**64**	cd599	KCNK2	27	309	309	180
**65**	cd604	THADA	28	660	660	123
**66**	cd608	ABCC10	23	237	228	117
**67**	cd609	ABCC10	25	480	471	114
**68**	cd616	CEBPZ	28	1122	1119	126
**69**	cd623	NUP13	26	222	222	120
**70**	cd624	GPER1	27	933	906	126
**71**	cd627	EPM2A	24	240	237	120
**72**	cd636	JMJD6	27	387	384	201
**73**	cd655	NSDHL	27	318	315	120
**74**	cd660	NUDT6	28	345	345	135
**75**	cd676	FRAS1	28	345	345	126
**76**	cd677	MOSPD1	25	219	216	120
**77**	cd687	ANKRD50	25	456	450	120
**78**	cd693	KIAA1239	28	591	591	150
**79**	cd695	FRAS1	25	201	201	126
**80**	cd698	PRDM8	26	228	228	111

	# CDS	External name	# Sequences in alignment	Query Sequence Length (bp)	Max Sequence Length captured (bp)	Min Sequence Length captured (bp)
**81**	cd699	FGF5	28	348	348	123
**82**	cd714	COPB1	25	225	225	78
**83**	cd722	RAG2	28	1419	1407	354
**84**	cd725	MYBPC3	27	258	258	108
**85**	cd727	DNAH10	27	420	417	144
**86**	cd758	PCNXL4	27	690	687	291
**87**	cd759	LRRC9	24	204	204	114
**88**	cd774	CDH23	13	210	204	147
**89**	cd805	DST	23	285	285	132
**90**	cd807	DTD1	28	234	234	123
**91**	cd814	FBXL4	28	498	498	129
**92**	cd817	RELN	26	231	228	87
**93**	cd834	TTN	28	300	300	105
**94**	cd845	RAB3GAP1	26	225	225	105
**95**	cd847	WDSUB1	28	387	381	132
**96**	cd850	PHKB	26	195	192	114
**97**	cd853	TSHZ3	26	255	255	126
**98**	cd856	TRIP4	26	198	198	105
**99**	cd880	SELRC1	27	399	396	126
**100**	cd888	FUT10	28	834	834	279
**101**	cd898	HMCN1	26	252	249	108
**102**	cd899	TRMT1L	27	300	252	150
**103**	cd905	IKBKAP	25	231	228	117
**104**	cd909	TRPC1	27	336	336	204
**105**	cd919	GPR171	28	870	870	177
**106**	cd927	TTN	28	273	273	126
**107**	cd932	TSPEAR	27	222	222	111
**108**	cd947	FYN	27	243	243	117
**109**	cd950	GLCE	25	231	231	129
**110**	cd953	ABCB7	27	210	210	90
**111**	cd960	CCDC127	26	636	636	126
**112**	cd963	NYX	27	915	915	270
**113**	cd971	DHTKD1	24	267	267	123
**114**	cd992	PDPR	27	399	399	126
**115**	cd998	FOCAD	26	234	234	117
**116**	cd1004	PIGM	26	255	255	105
**117**	cd1055	AHI1	26	186	183	120
**118**	cd1058	TNKS	26	237	237	57
**119**	cd1061	DPYD	25	213	213	123
**120**	cd1074	SLC35D3	28	435	432	114
**121**	cd1076	TRAPPC12	26	240	240	126
**122**	cd1078	RECQL5	18	468	468	174

	# CDS	External name	# Sequences in alignment	Query Sequence Length (bp)	Max Sequence Length captured (bp)	Min Sequence Length captured (bp)
**123**	cd1085	SORL1	23	243	243	171
**124**	cd1090	AGRN	25	213	210	120
**125**	cd1091	SLC22A23	20	291	288	135
**126**	cd1094	RAI2	26	315	303	192
**127**	cd1096	CLIP4	23	180	174	117
**128**	cd1099	LRP2	27	270	270	129
**129**	cd1108	GPR75	28	1503	1209	252
**130**	cd1110	SWAP70	25	225	225	117
**131**	cd1115	TUBGCP5	26	237	234	105
**132**	cd1123	NRDE2	25	312	312	123
**133**	cd1126	OXSM	28	855	855	219
**134**	cd1127	RAD50	14	195	192	117
**135**	cd1130	CCDC51	27	297	297	84
**136**	cd1133	ZNF598	26	219	219	123
**137**	cd1134	ASCC3	25	222	222	57
**138**	cd1145	HEPACAM	26	330	330	126
**139**	cd1150	HSPG2	25	264	264	105
**140**	cd1161	DNAH7	26	327	327	111
**141**	cd1171	DNAH5	27	321	318	159
**142**	cd1175	SLC35F6	28	372	372	123
**143**	cd1180	PID1	28	465	456	162
**144**	cd1195	EFHB	20	210	210	135
**145**	cd1201	VASH1	27	309	306	165
**146**	cd1207	KIAA1239	28	585	585	123
**147**	cd1217	ZFPM2	28	2142	1797	411
**148**	cd1234	SLC20A2	27	561	558	144
**149**	cd1239	USP19	25	207	207	126
**150**	cd1257	C8B	17	174	171	117
**151**	cd1258	KCTD4	28	726	726	123
**152**	cd1272	SRBD1	28	282	282	114
**153**	cd1279	MED23	28	246	246	114
**154**	cd1299	PDGFRL	27	234	231	120
**155**	cd1338	NBAS	23	228	228	117
**156**	cd1349	KLHL38	24	270	270	105
**157**	cd1351	KL	28	1116	1113	102
**158**	cd1352	KL	27	384	381	153
**159**	cd1354	VPS13C	21	387	387	90
**160**	cd1360	CPOX	22	216	216	111
**161**	cd1368	AIFM2	22	183	180	90
**162**	cd1371	KIAA1109	24	210	210	117
**163**	cd1374	SLC30A5	28	297	282	123
**164**	cd1379	LRIT3	23	255	255	120

	# CDS	External name	# Sequences in alignment	Query Sequence Length (bp)	Max Sequence Length captured (bp)	Min Sequence Length captured (bp)
**165**	cd1385	RAB30	26	237	237	126
**166**	cd1391	RGS9BP	28	543	543	264
**167**	cd1396	UBIAD1	21	411	408	78
**168**	cd1405	EOMES	28	582	561	171
**169**	cd1428	NARS	23	219	219	75
**170**	cd1429	DMRT3	28	837	837	291
**171**	cd1431	DAPK1	28	1518	1233	351
**172**	cd1438	GPR98	28	474	375	141

Table S3B: data missing per taxon in relation to the query sequence.

Genus	Species	Sequence length of concatenated 172 loci (linear unaligned, basepairs)	missing data
**Query:** ***Callorhinchus***	*milii*	71,234	0,00%
Squalus	*acanthias*	55,503	22,08%
Cirrhigaleus	*asper*	62,768	11,88%
Centrophorus	*moluccensis*	48,343	32,13%
Deania	sp.	22,732	68,09%
Dalatias	*licha*	45,206	36,54%
Isistius	*brasiliensis*	42,563	40,25%
Squaliolus	*aliae*	51,961	27,06%
Euprotomicrus	*bispinatus*	56,696	20,41%
Somniosus	*microcephalus*	44,741	37,19%
Zameus	*squamulosus*	49,333	30,75%
Scymnodon	*ringens*	43,818	38,49%
Centroselachus	*crepidater*	46,804	34,30%
Centroscymnus	*coelolepis*	52,123	26,83%

Scymnodon	*plunketi*	50,336	29,34%
Oxynotus	*paradoxus*	49,411	30,64%
Aculeola	*nigra*	51,910	27,13%
Centroscyllium	*fabricii*	59,249	16,82%
Etmopterus	*splendidus*	48,886	31,37%
Etmopterus	*sheikoi*	64,262	9,79%
Etmopterus	cf. *molleri*	57,800	18,86%
Etmopterus	*spinax*	50,153	29,59%
Etmopterus	*gracilispinis*	49,907	29,94%
Trigonognathus	*kabeyai*	61,048	14,30%
Echinorhinus	*brucus*	40,248	43,50%
Pliotrema	*warreni*	58,028	18,54%
Pristiophorus	*japonicus*	56,426	20,79%
Squatina	*nebulosa*	55,156	22,57%
Hexanchus	*griseus*	51,674	27,46%

Table S4: Overview of different phylogeentic analyses performed on the concatenated 172 loci dataset

Dataset	Method	Tool	Substitution Model	Partition scheme	Outgroup
**Concatenated 1265 cds nucleotide loci**	Maximum likelihood	RAxML	GTRCAT	none	*Hexanchus griseus*
**Concatenated 1265 cds nucleotide loci**	Maximum likelihood	RAxML	GTR+G	$1^{st}+2^{nd}$ & 3rd codon	*Hexanchus griseus*
**Concatenated 1265 cds nucleotide loci**	Maximum likelihood	RAxML	GTR+G	By gene	*Hexanchus griseus*
**MARE reduced 174 loci amino acid matrix**	Maximum likelihood	RAxML	JTT+G	By gene	*Hexanchus griseus*
**MARE reduced 174 loci amino acid matrix**	Maximum likelihood	RAxML	Blosum62	By gene	*Hexanchus griseus*
**MARE reduced 174 loci amino acid matrix**	Maximum likelihood	RAxML	see partition file*	Best partition scheme suggested by partitionfinderProtei n vers. 1.1.1*	*Hexanchus griseus*
**Concatenated 172 cds nucleotide loci**	Maximum likelihood	RAxML	GTRCAT	none	*Hexanchus griseus*
**Concatenated 172 cds nucleotide loci**	Maximum likelihood	RAxML	GTR+G	$1^{st}+2^{nd}$ & 3rd codon	*no outgroup defined*
**Concatenated 172 cds nucleotide loci**	Maximum likelihood	RAxML	GTR+G	by gene and $1^{st}+2^{nd}$ & 3rd codon	*Hexanchus griseus*
**Concatenated 172 cds nucleotide loci**	Maximum likelihood	RAxML	see partition file**	Best partition scheme suggested by partitionfinder vers. 1.1.1**	*Hexanchus griseus*
**Concatenated 172 cds nucleotide loci**	Maximum likelihood	RAxML	GTRCAT	none	no outgroup defined
**Concatenated 172 cds nucleotide loci**	Maximum likelihood	RAxML	GTRCAT	none	no outgroup defined, *Hexanchus griseus* deleted from alignment
**Concatenated 172 amino acid loci**	Bayesian	PhyloBayes	CAT	none	n.a.
**Concatenated 172 cds nucleotide loci**	Bayesian inference	BEAST	GTR+G	$1^{st}+2^{nd}$ & 3rd codon	*Hexanchus griseus*

*PartitionfinderProtein best partitioning scheme for RAxML:

branchlengths : linked
models : MTMAM+G+F, MTMAM+G, DAYHOFF+G, DCMUT+G+F, VT+G+F, DCMUT+G, JTT+G, LG+G+F, LG+G, MTREV+G, MTREV+G+F, RTREV+G+F, WAG+G, CPREV+G, CPREV+G+F, WAG+G+F, JTT+G+F, DAYHOFF+G+F, RTREV+G, VT+G, BLOSUM62+G, BLOSUM62+G+F
model_selection : bic
search : rcluster
weights : rate = 1, base = 0, model = 0, alpha = 0
rcluster-percent : 1.0

JTT, p1 = 1-285, 1188-1477, 2030-2206, 2803-3004, 3521-3606, 5044-5593, 6280-6495, 8078-8206, 11480-11556, 11997-12071, 12072-12195, 12818-12898, 13178-13335, 15183-15256, 16167-16538, 16618-16726, 16727-16965, 17816-17930, 19078-19169, 20052-20135, 20743-20813, 20888-21129, 21744-21811, 22612-22748, 22749-22877, 23174-23254, 24016-24095, 24096-24166
JTT, p2 = 286-381, 382-686, 3170-3248, 6210-6279, 13336-13457
JTT, p3 = 687-1187, 11871-11996
JTT, p4 = 1478-1661
JTT, p5 = 1662-1756, 1869-2029, 3249-3338, 13701-13840, 23662-23740
JTT, p6 = 1757-1868, 14491-14685, 15969-16041, 18332-18404, 19423-19499, 21517-21743, 22401-22499, 22500-22611
JTT, p7 = 2207-2436, 6103-6209, 7642-7793, 8207-8920, 10152-10279, 11713-11797, 11798-11870, 18466-18535, 19170-19234, 19828-19902, 20269-20413, 21423-21516
MTMAM, p8 = 2437-2536
JTT, p9 = 2537-2733, 4119-4416, 7300-7388, 13458-13527
JTT, p10 = 2734-2802, 10535-10638, 11625-11712
JTT, p11 = 3005-3095
JTT, p12 = 3096-3169, 3607-3826, 8001-8077, 9837-9913, 10435-10534, 11353-11479, 15423-15501, 15903-15968, 21130-21219
JTT, p13 = 3339-3520
JTT, p14 = 3827-3901
JTT, p15 = 3902-3967, 10280-10365, 20511-20742, 23417-23506
JTT, p16 = 3968-4118, 11267-11352, 12662-12817, 17931-18142
JTT, p17 = 4417-4496, 6035-6102, 12984-13177, 14058-14182, 16539-16617, 18227-18331, 19308-19422, 23255-23336, 23337-23416
JTT, p18 = 4497-4566, 11557-11624, 14260-14365, 14964-15037, 15257-15422, 16042-16166, 18143-18226, 21883-22159, 22291-22400, 23032-23097
JTT, p19 = 4567-4784, 14366-14428
JTT, p20 = 4785-4883, 5594-5967, 10082-10151, 12899-12983, 13528-13700, 14429-14490, 15111-15182

JTT, p21 = 4884-5043
JTT, p22 = 5968-6034, 7794-8000, 9979-10081, 16966-17471, 18612-18890, 18891-19077, 19750-19827, 19903-19978, 20414-20510, 20814-20887, 22218-22290, 23741-23937, 23938-24015
JTT, p23 = 6496-6971, 6972-7112, 8921-9053, 9914-9978, 12351-12661, 13841-13914, 14686-14963, 17640-17755, 17756-17815, 21320-21422, 22878-22952
JTT, p24 = 7113-7299, 9054-9131, 13990-14057, 18536-18611, 21220-21319
JTT, p25 = 7389-7483, 9132-9260, 9261-9836, 17472-17639, 19235-19307, 19500-19749, 20136-20268, 23507-23661, 24167-24235, 24236-24461
JTT, p26 = 7484-7641, 10726-11198
JTT, p27 = 10366-10434
JTT, p28 = 10639-10725, 14183-14259, 15038-15110, 23098-23173
JTT, p29 = 11199-11266, 12281-12350, 22160-22217
MTMAM, p30 = 12196-12280
JTT, p31 = 13915-13989, 15502-15902
JTT, p32 = 18405-18465
JTT, p33 = 19979-20051
MTREV, p34 = 21812-21882
JTT, p35 = 22953-23031

**Partitionfinder best partitioning scheme for RAxML:

branchlengths : linked
models : GTR+I+G, GTR+G
model_selection : bic
search : rcluster
weights : rate = 1, base = 0, model = 0, alpha = 0
rcluster-percent : 0.1

DNA, p1 = 1-453, 6391-6612, 9164-9541, 32430-32669, 32670-33056, 43575-43826, 51471-51683, 51684-51974, 56583-56804, 56805-57134
DNA, p2 = 454-1881, 2563-2751, 3919-4170, 4603-4815, 5026-5508, 5740-5967, 5968-6390, 6913-7116, 7117-7320, 7525-7749, 8008-8265, 10595-11185, 11186-11560, 11768-12043, 12263-12466, 12674-13204, 13205-14407, 15935-16495, 16496-17116, 17621-19270, 19271-19876, 21368-21565, 21566-22111, 22112-22396, 22667-22897, 23264-23644, 25613-25931, 26586-27335, 27336-28229, 28230-28466, 28776-29435, 30153-31274, 31275-31496, 33057-33374, 33375-33719, 33720-34064, 34065-34283, 34284-34739, 34740-35330, 35331-35531, 35760-36107, 36333-37751, 38430-39119, 39324-39533, 39819-40052, 40782-41081, 41082-41306, 41694-41888, 41889-42143, 42144-42341, 42342-42740, 42741-43574, 43827-44126, 44127-44357, 44358-44693, 44694-45563,

45837-46058, 46302-46532, 46533-46742, 46743-47378, 48960-49193, 49194-49448, 49449-49634, 49635-49871, 49872-50084, 50085-50519, 50520-50759, 52290-52469, 52470-52739, 54468-54704, 55017-55871, 55872-56066, 56067-56363, 56364-56582, 57726-58046, 58419-58883, 59094-59402, 59403-59987, 59988-62129, 62691-62897, 62898-63071, 63072-63797, 63798-64079, 64326-64559, 64560-64787, 64788-65057, 65058-66173, 66174-66557, 66945-67160, 67161-67343, 67344-67553, 67554-67850, 67851-68105, 69297-69878, 72453-72926
DNA, p3 = 1882-2562, 4171-4392, 4393-4602, 4816-5025, 5509-5739, 6613-6912, 8266-8463, 8464-8689, 8690-9163, 9542-10219, 11561-11767, 12467-12673, 14408-15103, 19877-20593, 20594-20929, 21149-21367, 22397-22666, 22898-23263, 23645-25372, 25373-25612, 25932-26585, 29436-29672, 29673-30152, 31497-32429, 36108-36332, 37752-38009, 38010-38429, 39534-39818, 40053-40550, 45564-45836, 46059-46301, 47379-48293, 48294-48560, 48561-48959, 50760-51227, 51228-51470, 54243-54467, 54705-55016, 57135-57398, 57399-57725, 58047-58418, 58884-59093, 62130-62690, 64080-64325, 66558-66944, 68886-69296
DNA, p4 = 2752-3399, 3400-3918, 7750-8007, 20930-21148, 52740-54242
DNA, p5 = 7321-7524, 10220-10594, 12044-12262, 15104-15934, 17117-17620, 28467-28775, 35532-35759, 39120-39323, 40551-40781, 41307-41693, 51975-52289, 68106-68342, 69879-70097, 70098-70934, 70935-72452
DNA, p6 = 68343-68885

Outgroup testing

For testing the influence of different outgroups, we compared unrooted topologies (Maximum Likelihood, computed with RAxML under GTRCAT).

a)

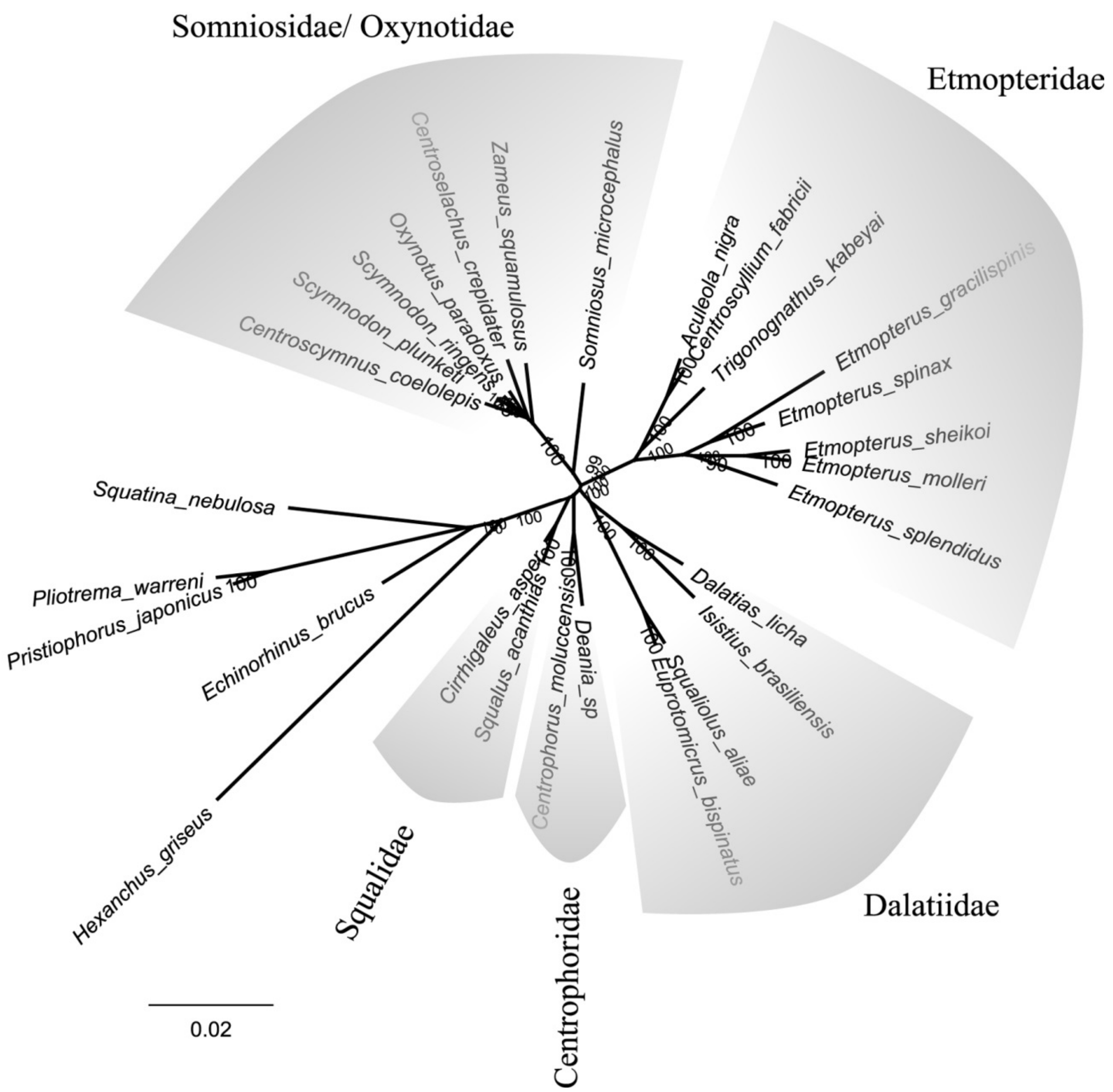

b)

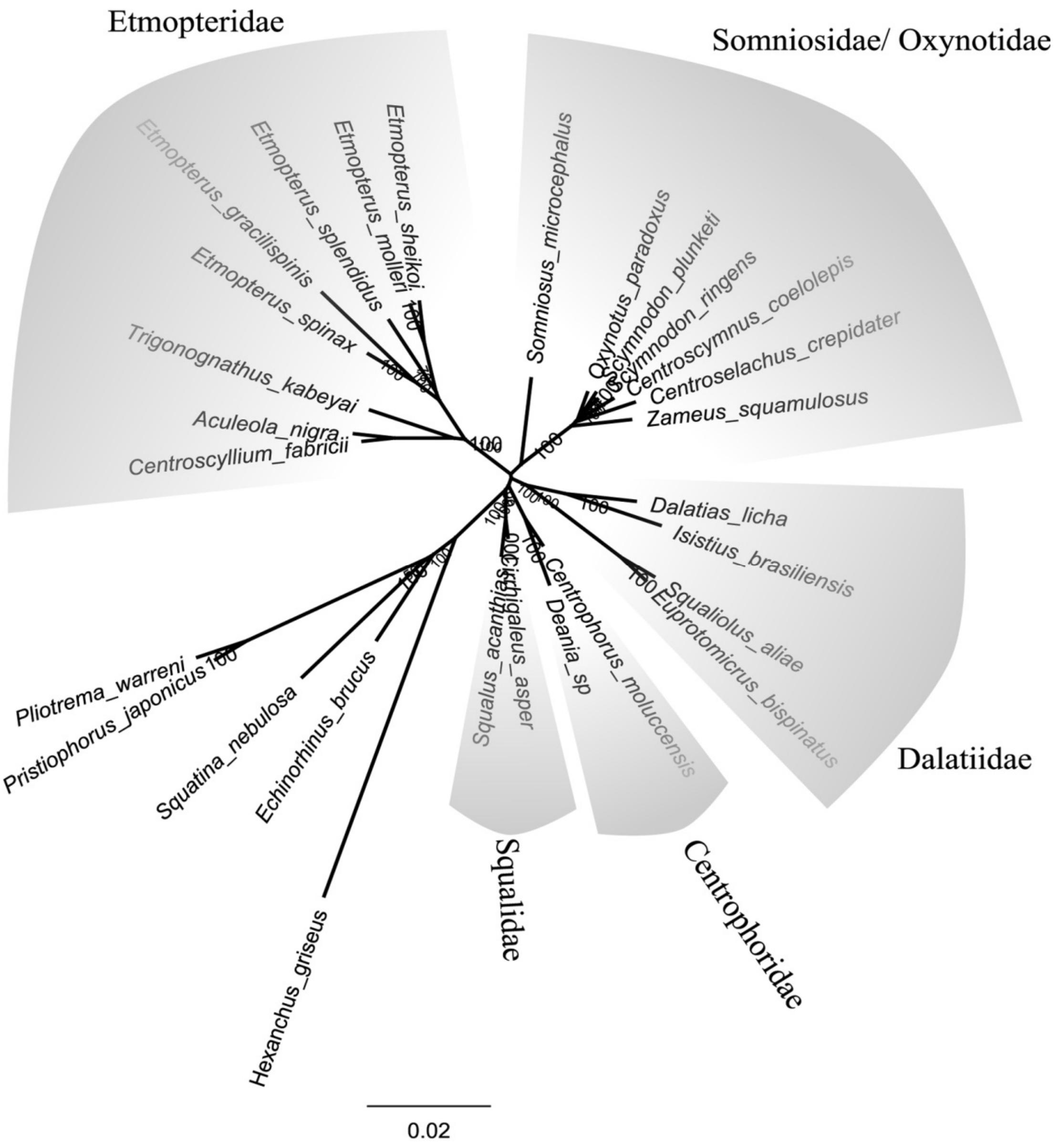
Etmopteridae
Somniosidae/ Oxynotidae
Dalatiidae
Centrophoridae
Squalidae
Etmopterus_gracilispinis
Etmopterus_splendidus
Etmopterus_molleri
Etmopterus_sheikoi
Etmopterus_spinax
Trigonognathus_kabeyai
Aculeola_nigra
Centroscyllium_fabricii
Somniosus_microcephalus
Oxynotus_paradoxus
Centroscymnus_coelolepis
Centroselachus_crepidater
Zameus_squamulosus
Dalatias_licha
Isistius_brasiliensis
Squaliolus_aliae
Euprotomicrus_bispinatus
Centrophorus_moluccensis
Deania_sp
Cirrhigaleus_asper
Squalus_acanthias
Pliotrema_warreni
Pristiophorus_japonicus
Squatina_nebulosa
Echinorhinus_brucus
Hexanchus_griseus
0.02

c)

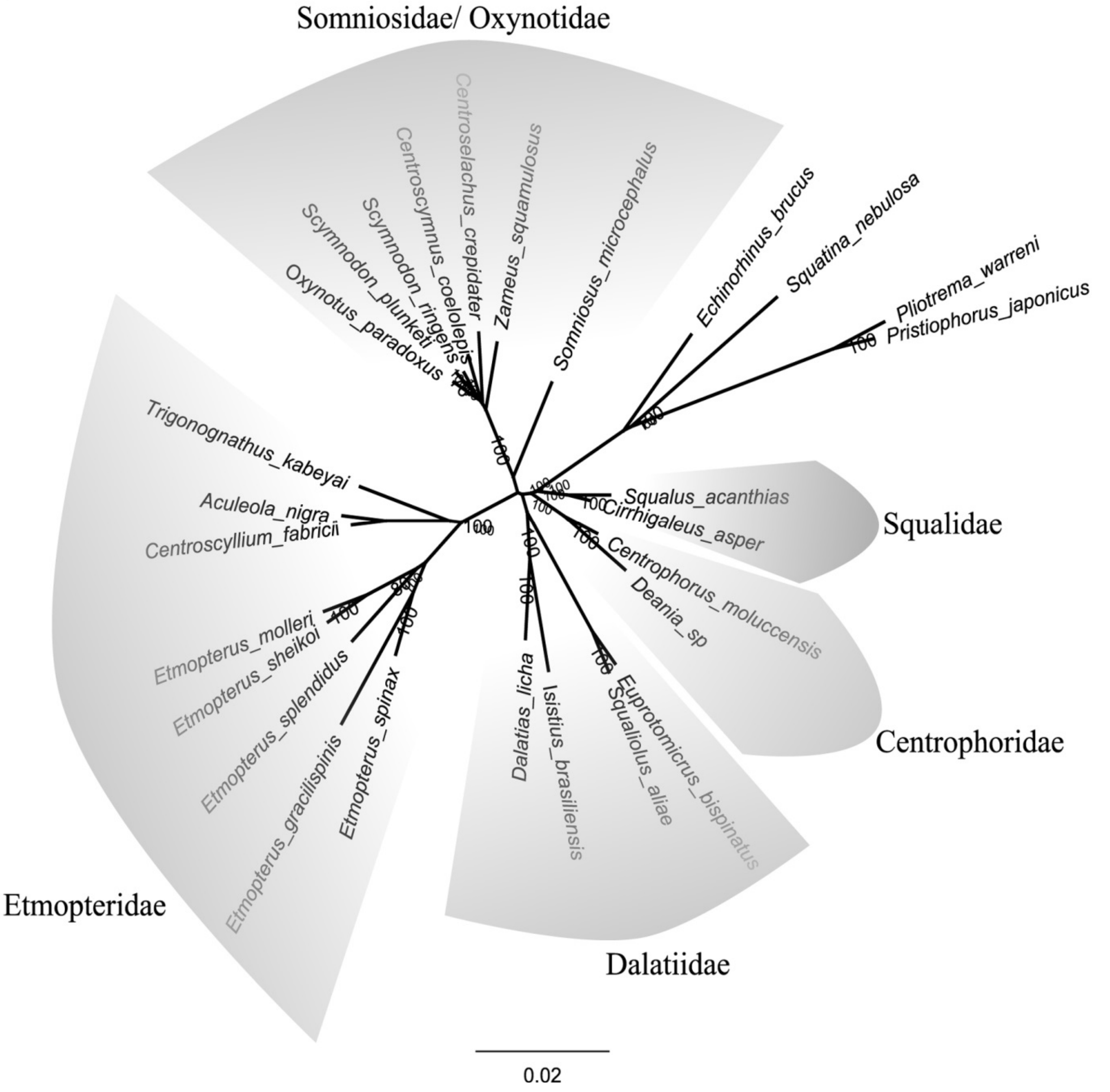
Somniosidae/ Oxynotidae
Centroselachus_crepidater
Zameus_squamulosus
Centroscymnus_coelolepis
Scymnodon_ringens
Scymnodon_plunketi
Oxynotus_paradoxus
Somniosus_microcephalus
Echinorhinus_brucus
Squatina_nebulosa
Pliotrema_warreni
Pristiophorus_japonicus
Trigonognathus_kabeyai
Aculeola_nigra
Centroscyllium_fabricii
Squalus_acanthias
Cirrhigaleus_asper
Squalidae
Centrophorus_moluccensis
Deania_sp
Centrophoridae
Etmopterus_molleri
Etmopterus_sheikoi
Etmopterus_splendidus
Etmopterus_gracilispinis
Etmopterus_spinax
Dalatias_licha
Isistius_brasiliensis
Squaliolus_aliae
Euprotomicrus_bispinatus
Etmopteridae
Dalatiidae
0.02

d)

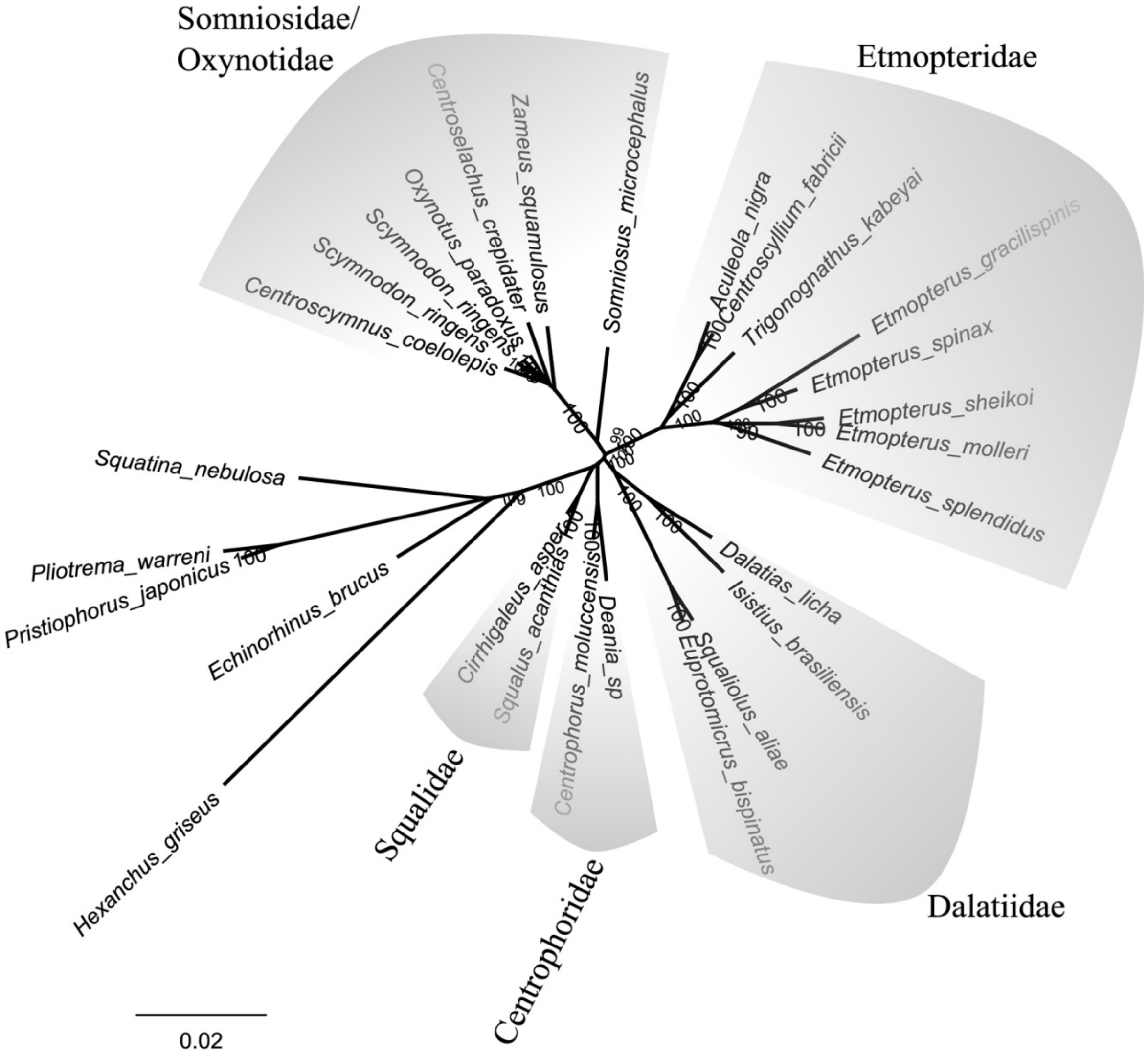

Figure S2: Maximum likelihood analyses comparing based on 172 nucleotide loci. Tree computed using RAxML [35]under GTRCAT.

a) *Hexanchus griseus* chosen as outgroup.

b) no outgroup defined

c) excluding *Hexanchus griseus* from the analysis, no outgroup defined

d) defining *Pristiophurus japonicus* and *Pliotrema warreni* as outgroup taxa.

Phylogenetic analysis:

Resulting phylogenetic estimates from analyses described in Table S4:

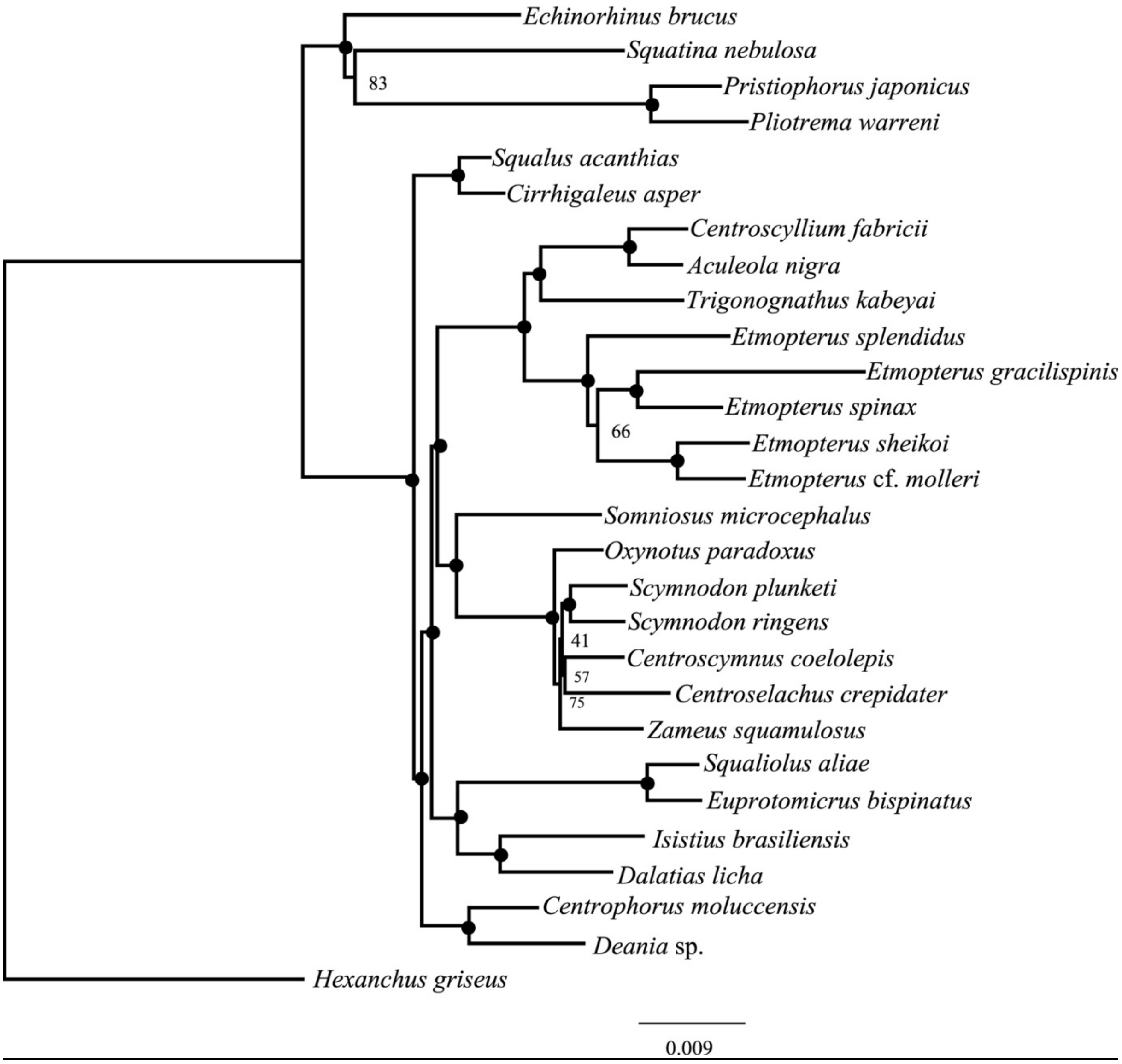

Figure S3: Maximum likelihood analysis using RAxML [35] under a GTRGAMMA model based on the full nucleotide dataset of 1265 loci including all sequence information. Data partitioned by 1st, 2nd and 3rd codon position. Black dots indicate 100% bootstrap support from 100 bootstrap replicates drawn. *Hexanchus griseus* was defined as outgroup.

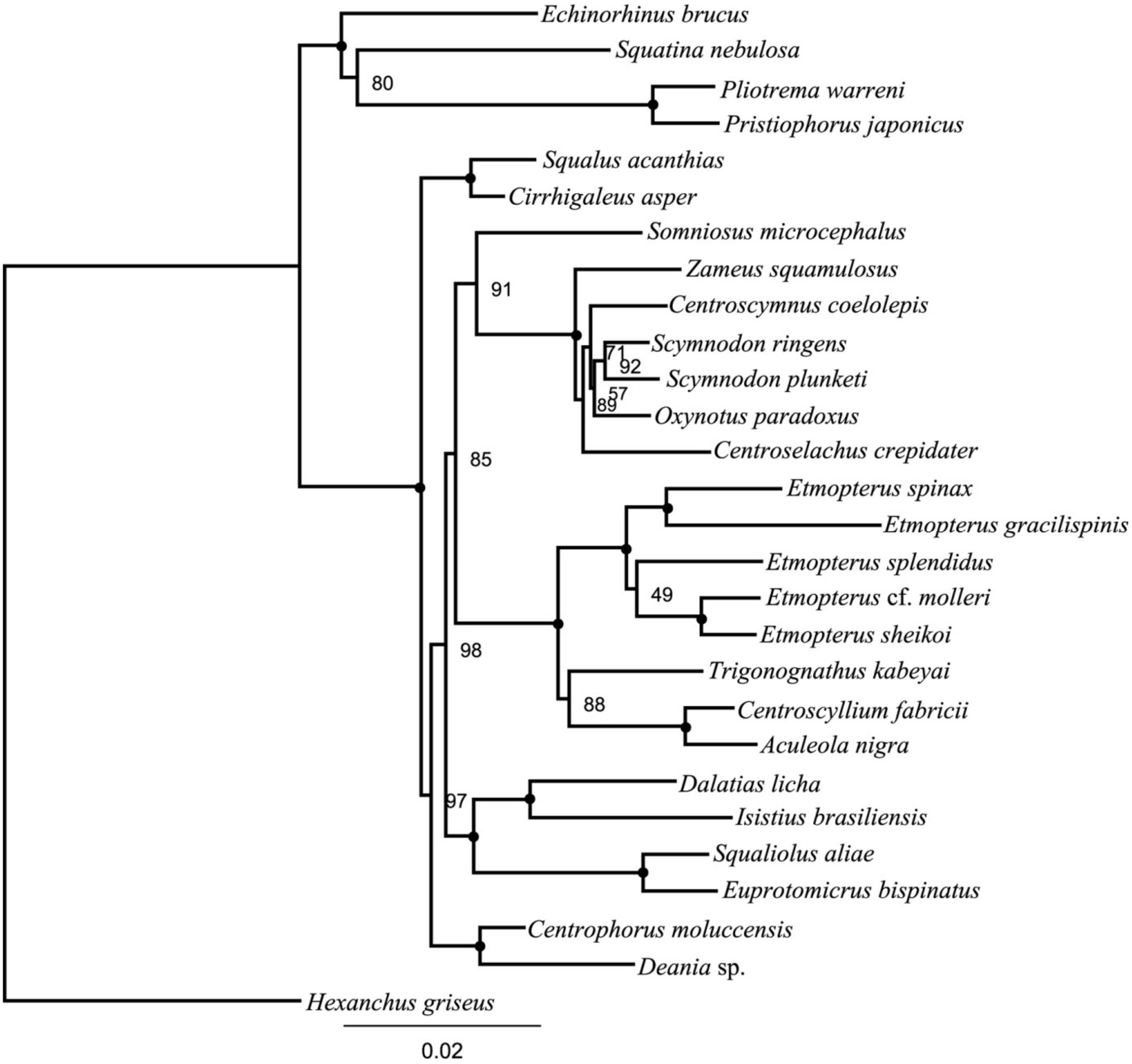

Figure S4: Maximum likelihood analysis using RAxML [35] based on the 174 loci translated to amino acids resulting from the MARE [33, 34]analysis. Data partition and model applications are based on the result of Partitionfinder Protein [62, 63] applied to the data before the ML analysis (see Table 3 for details on the partitioning scheme). Black dots indicate 100% bootstrap support from 100 bootstrap replicates drawn. *Hexanchus griseus* was defined as outgroup.

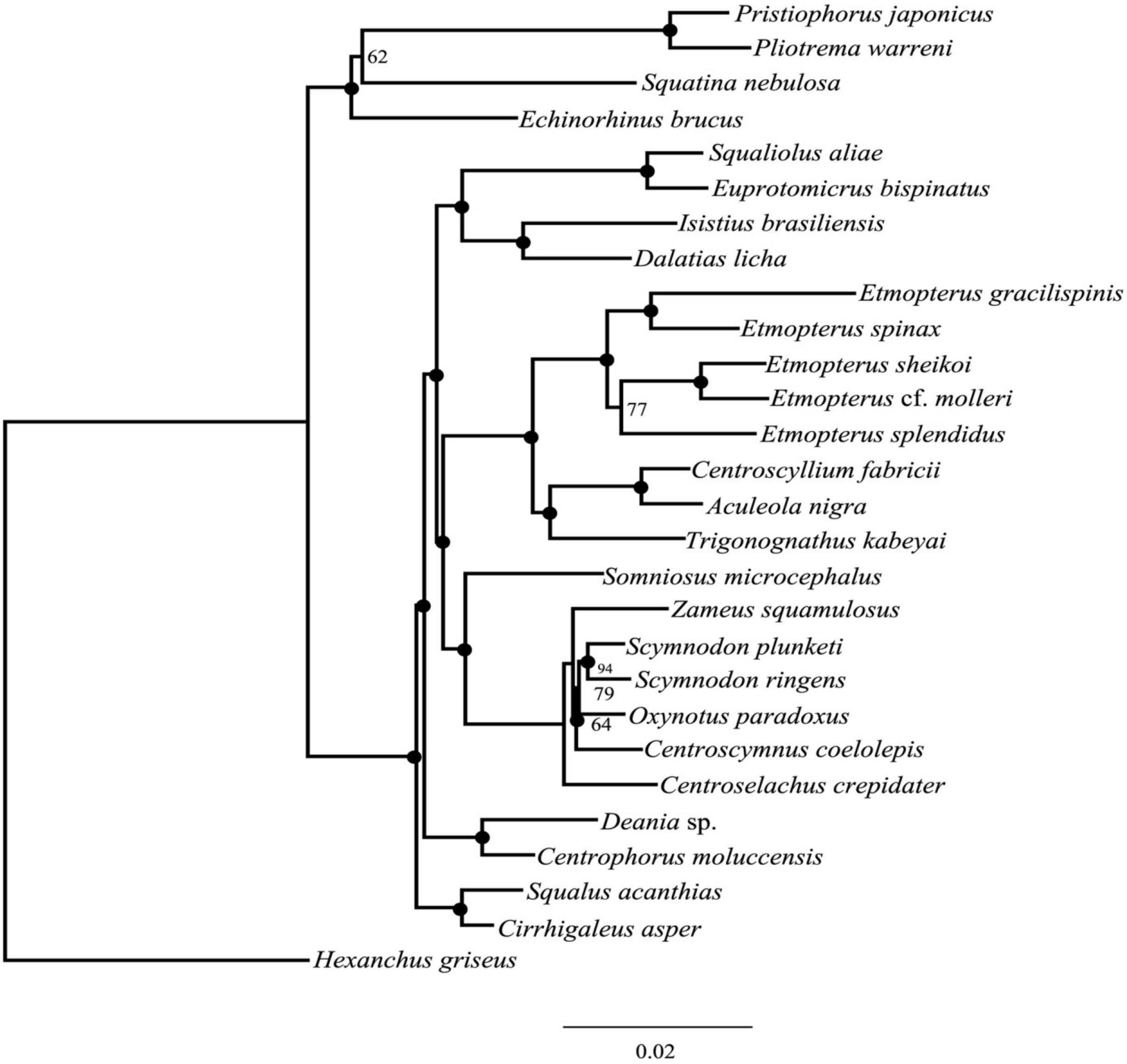

Figure S5: Maximum likelihood analysis using RAxML [35] based on the 172 loci translated to nucleotides resulting from the MARE [33, 34]and re-blast analysis. The data partition is based on the result of Partitionfinder [62, 63] applied to the data (see Table S4 for details on the partitioning scheme). Black dots indicate 100% bootstrap support from 100 bootstrap replicates drawn. *Hexanchus griseus* was defined as outgroup.

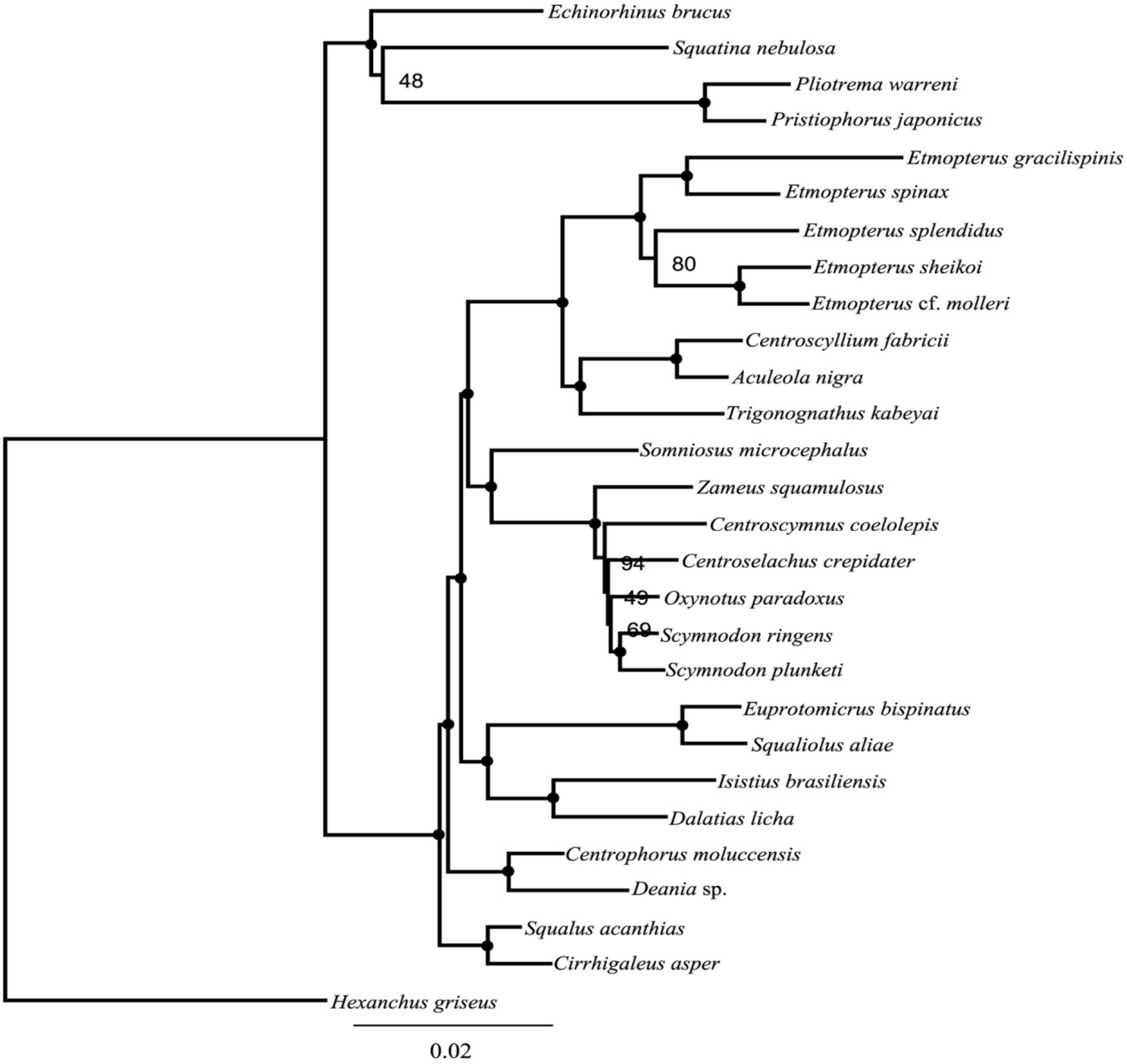

Figure S6: Maximum likelihood analysis using RAxML [35] based on the 172 loci translated to nucleotides resulting from the MARE and re-blast analysis. GTR GAMMA is used on unpartitioned data. Black dots indicate 100% bootstrap support from 100 bootstrap replicates drawn. *Hexanchus griseus* was defined as outgroup.

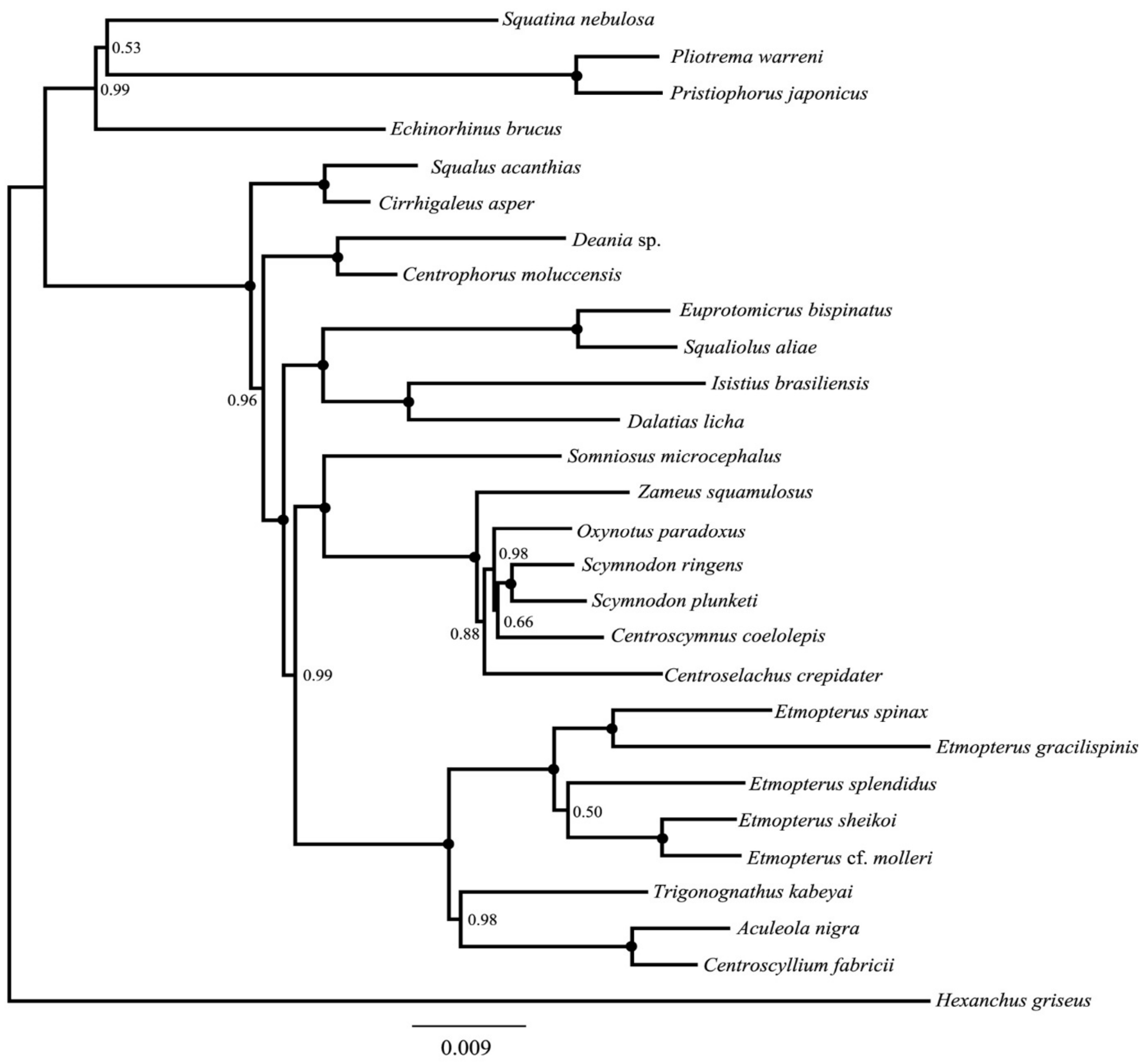

Figure S7: Bayesian inference using PhyloBayes 3.3f [36, 37] based on the 172 amino acid loci resulting from the MARE and re-blast analysis. The CAT model [64] is used on the unpartitioned dataset. Four chains were run in parallel. The tracefiles and treelists of all four chains were used to check for convergence . The analysis was stopped with a maximum difference of 0.16 and effective sample sizes exceeding 100, with the exception of the allocent statistic (see analysis protocol below). A majority rule consensus tree was computed from 12997 input trees from each chain (burnin = 1000 trees, analyzing every second tree of the pooled trees). Black dots indicate posterior probabilities of 1. Tree midpoint-rooted using Figtree 1.4.2 [69].

Analysis protocol for the Bayesian inference using Phylobayes 3.3f [36, 37]:

Phylobayes was called as follows:

pb -d "input alignmnet of 172 aminoacid loci" -cat -nchain 4 500 0.1 100.

tracecomp output after 4734362 generations:

name effsize rel_diff

loglik	448	0.139033
length	3877	0.0473159
alpha	831	0.106427
nocc	410	0.163083
stat	457	0.111566
statalpha	19448	0.026536
kappa	881	0.103027
allocent	52	0.499115

bpcomp output after 4734362 generations:

maxdiff: 0.157806

meandiff: 0.00259295

Plot of log-likelihoods for 4734362 generations:

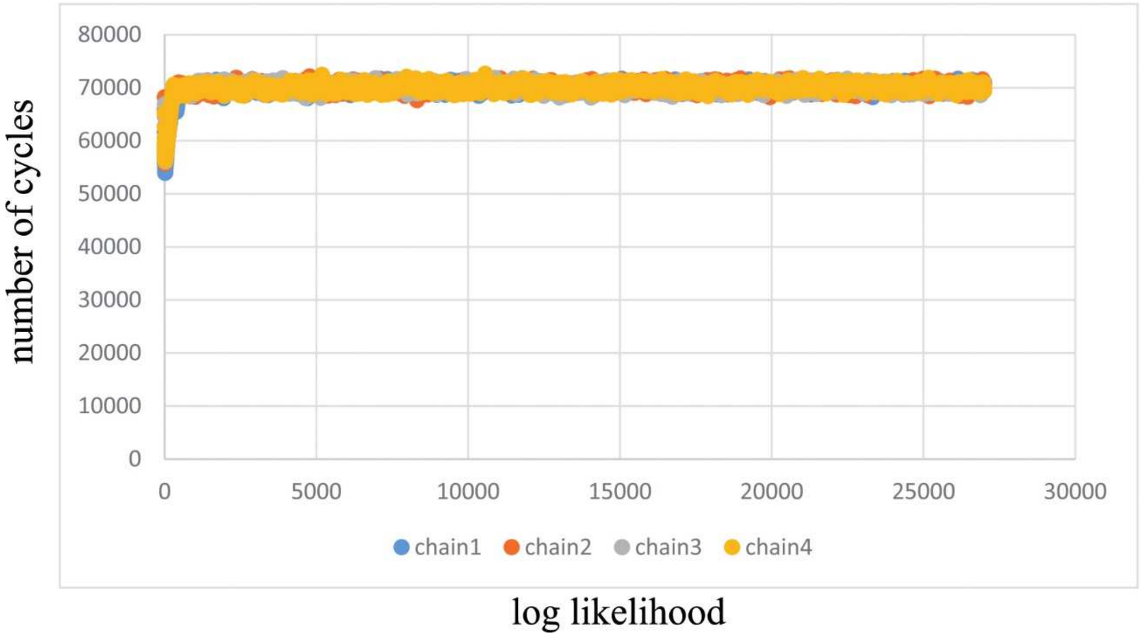

<u>Node time estimation using BEAST</u>

BEAUTi was used to create XML formatted input files for BEAST. Please see Table 2 in the main text for calibration points used. The full XML input file is available at http://datadryad.org/review?doi=doi:10.5061/dryad.n3581

MEDUSA [42, 43] analysis for estimating diversification rates and shifts

Table S5: species richness table

taxon	Species richness values (deployed from [1])
Aculeola	1
Centrophorus	12
Centroselachus	1
Centroscyllium	7
Centroscymnus	3
Cirrhigaleus	3
Dalatias	1
Deania	4
Echinorhinus	2
***Etmopterus lucifer* clade**	11
***Etmopterus gracilispinis* clade**	7
Etmopterus sheikoi	1
***Etmopterus spinax* clade**	10
***Etmopterus pusillus* clade**	8
Euprotomicrus	1
Hexanchus	2
Isistius	2
Oxynotus	5
Pliotrema	1
Pristiophorus	7
Scymnodon plunketi	1
Scymnodon	3
Somniosus	4
Squalus	25
Squaliolus	2
Squatina	22
Trigonognathus	1
Zameus	1

Note that *Etmopterus villosus* was not included in the analysis, as its *Etmopterus* subclade designation is unclear [23].

R protocol:

```
R version 3.0.3 (2014-03-06) -- "Warm Puppy"

Copyright (C) 2014 The R Foundation for Statistical Computing

Platform: x86_64-w64-mingw32/x64 (64-bit)

> library (laser)
Lade nötiges Paket: ape
Lade nötiges Paket: geiger

#loading timetree estimated with BEAST.
>  read.tree ("172.tre")->tree
> tree
```

```
Phylogenetic tree with 28 tips and 27 internal nodes.

Tip labels:
       Centrophorus_ moluccensis, Deania_sp, Etmopterus_gracilispinis, Etmopterus_spinax,
Etmopterus_molleri, Etmopterus_sheikoi, ...
Rooted; includes branch lengths.

#loading richness table including species richness information for each branch in the tree.
>  read.csv ("172richness_2.csv") ->richness
> richness
                      taxon ntaxa
1     Isistius_brasiliensis     2
2         Hexanchus_griseus     6
3            Dalatias_licha     1
4        Etmopterus_molleri    11
5  Centroselachus_crepidater     1
6     Trigonognathus_kabeyai     1
7    Centroscyllium_fabricii     7
8         Etmopterus_spinax    10
9        Etmopterus_sheikoi     1
10   Somniosus_microcephalus     4
11  Euprotomicrus_bispinatus     1
12         Pliotrema_warreni     1
13   Pristiophorus_japonicus     7
14        Cirrhigaleus_asper     3
15        Squalus_acanthias    25
16    Scymnodon_plunketi     1
17           Aculeola_nigra     1
18    Centroscymnus_coelolepis     3
19       Zameus_squamulosus     2
20        Squatina_nebulosa    22
21  Etmopterus_gracilispinis     7
22       Oxynotus_paradoxus     5
23        Scymnodon_ringens     3
24         Squaliolus_aliae     2
25   Centrophorus_moluccensis    12
26    Etmopterus_splendidus     8
27      Echinorhinus_brucus     2
28               Deania_sp     4

#starting MEDUSA, using AICc as stopping criterion
> res1=medusa(tree, richness, warnings=FALSE)
Appropriate AICc threshold for tree of 28 tips is: 1.091845.

Step 1: lnLik=-187.6063; aicc=379.4434; model=bd
Step 2: lnLik=-183.8979; aicc=376.5959; shift at node 23; model=yule; cut=stem; # shifts=1
Step 3: lnLik=-181.5832; aicc=374.391; shift at node 35; model=yule; cut=stem; # shifts=2

No significant increase in aicc score. Disregarding subsequent piecewise models.

  Model.ID Shift.Node Cut.At Model Ln.Lik.part         r epsilon     r.low    r.high
1        1         29   node  yule   -85.80499 0.0198648      NA 0.0147561 0.0263547
2        2         23   stem  yule   -4.198604 0.0841583      NA 0.0471845 0.1585100
3        3         35   stem  yule   -91.57965 0.0410316      NA 0.0308829 0.0535196
> print(names(res1)) # output list elements
```

```
[1] "control"      "cache"        "model"        "summary"      "zSummary"      "medusaVersion"
[7] "richness"
> print(res1$summary) # show 'summary' object
  Model.ID Shift.Node Cut.At Model Ln.Lik.part        r epsilon     r.low    r.high
1        1         29   node  yule   -85.80499 0.0198648      NA 0.0147561 0.0263547
2        2         23   stem  yule   -4.198604 0.0841583      NA 0.0471845 0.1585100
3        3         35   stem  yule   -91.57965 0.0410316      NA 0.0308829 0.0535196
> summary(res1, criterion="aicc")
              Length Class      Mode
control         3    -none-     list
cache           2    -none-     list
model          11    -none-     list
summary         9    data.frame list
zSummary      756    -none-     numeric
medusaVersion   1    -none-     numeric
richness        2    data.frame list

#plotting results, plot breakpoints for best AIC model (see GEIGER package manual)
> plot(res1, cex=0.5,label.offset=1, edge.width=2)
```

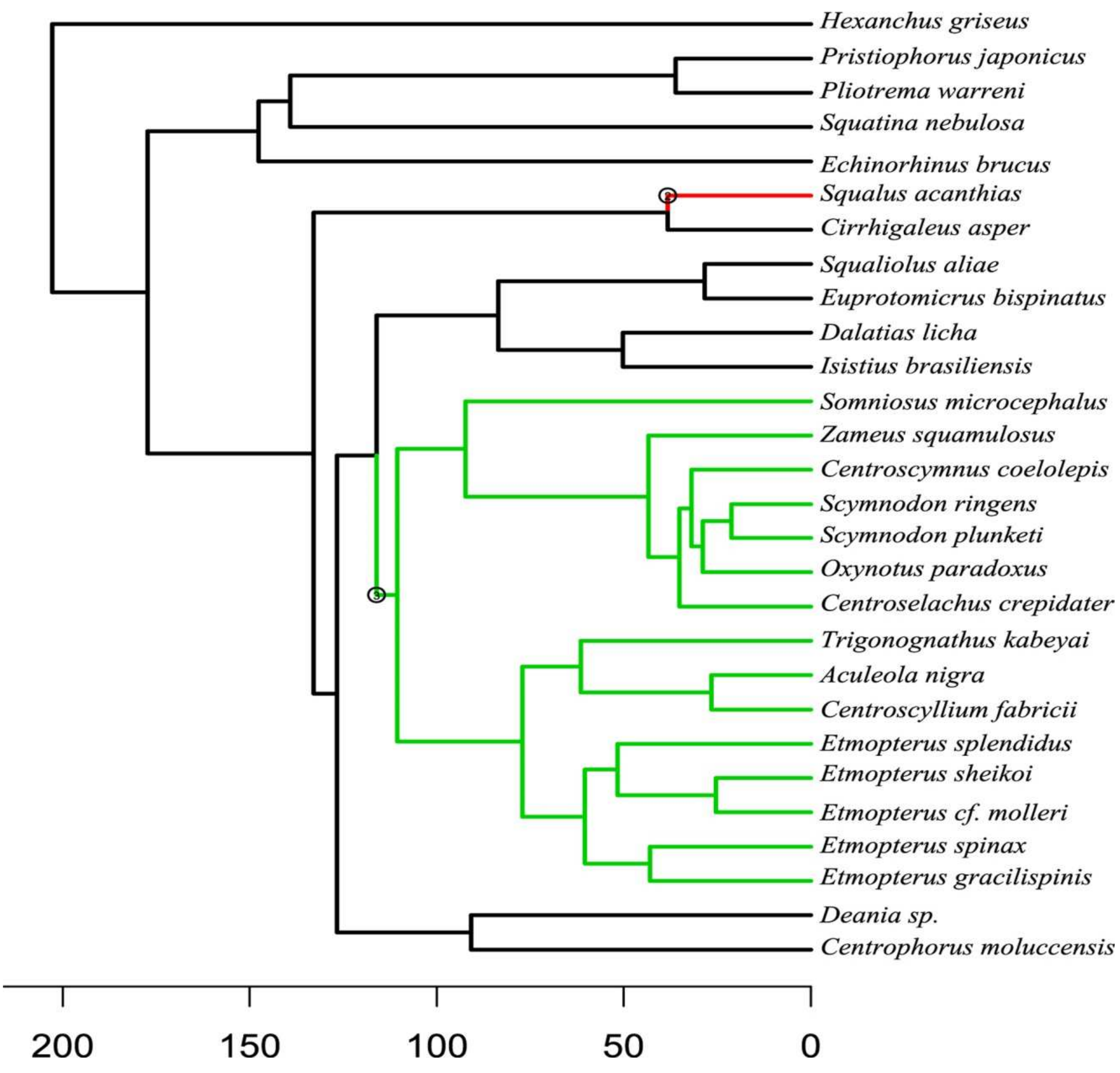

Figure S8: Output tree of MEDUSA [42, 43] analysis. Colored branches and dots mark significant increases in rate shifts, i.e. at the radiation of Etmopteridae and Somniosidae as well as the radiation of *Squalus*.

Species richness values were derived from the bibliographic database www.shark-references.com (accessed 18.04.2015) [1].

GEIGER Package [42] for ancestral character state reconstruction of occurrence of Bioluminescence

Table S6: Presence and absence of photophores in Squaliform sharks based on different information tested in the ancestral character state reconstructions.

taxon	Presence of photophores (0=absent, 1=present; assuming Somniosidae as non-luminous)	Presence of photophores (0=absent, 1=present; based on observations in this study)	Presence of photophores (0=absent, 1=present; based on [8])
Aculeola nigra	1	1	1
Centrophorus moluccensis	0	0	0
Centroselachus crepidater	0	0	1
Centroscyllium fabricii	1	1	1
Centroscymnus coelolepis	0	0	1
Cirrhigaleus asper	0	0	0
Dalatias licha	1	1	1
***Deania* sp.**	0	0	0
Echinorhinus brucus	0	0	0
Etmopterus* cf. *molleri	1	1	1
Etmopterus gracilispinis	1	1	1
Etmopterus sheikoi	1	1	1
Etmopterus spinax	1	1	1
Etmopterus splendidus	1	1	1
Euprotomicrus bispinatus	1	1	1
Hexanchus griseus	0	0	0
Isistius brasiliensis	1	1	1
Oxynotus paradoxus	0	0	0
Pliotrema warreni	0	0	0
Pristiophorus japonicus	0	0	0
Scymnodon plunketi	0	0	1
Scymnodon ringens	0	0	1
Somniosus microcephalus	0	0	0
Squalus acanthias	0	0	0
Squaliolus aliae	1	1	1
Squatina nebulosa	0	0	0

Trigonognathus kabeyai	1	1	1
Zameus squamulosus	0	1	1

R protocol:

```
R version 3.0.3 (2014-03-06) -- "Warm Puppy"
Copyright (C) 2014 The R Foundation for Statistical Computing
Platform: x86_64-w64-mingw32/x64 (64-bit)

> library (laser)

#loading different tables containing information on presence or absence of photphores in taxa.
#Lumi.csv= Table 4, column 1 as example.
> read.csv ("Lumi.csv")
                  taxon Lumi2
1      Isistius_brasiliensis     1    0
2         Hexanchus_griseus     0    0
3            Dalatias_licha     1    0
4        Etmopterus_molleri     1    0
5  Centroselachus_crepidater     0    0
6    Trigonognathus_kabeyai     1    0
7   Centroscyllium_fabricii     1    0
8         Etmopterus_spinax     1    0
9        Etmopterus_sheikoi     1    0
10   Somniosus_microcephalus     0    0
11  Euprotomicrus_bispinatus     1    0
12         Pliotrema_warreni     0    0
13   Pristiophorus_japonicus     0    0
14        Cirrhigaleus_asper     0    0
15         Squalus_acanthias     0    0
16      Scymnodon_plunketi     0    0
17            Aculeola_nigra     1    0
18    Centroscymnus_coelolepis     0    0
19       Zameus_squamulosus     0    0
20        Squatina_nebulosa     0    0
21  Etmopterus_gracilispinis     1    0
22       Oxynotus_paradoxus     0    0
23        Scymnodon_ringens     0    0
24          Squaliolus_aliae     1    0
25   Centrophorus_moluccensis     0    0
26    Etmopterus_splendidus     1    0
27       Echinorhinus_brucus     0    0
28               Deania_sp     0    0
> read.csv ("Lumi.csv") -> Lumi.dat
>   data.frame(Lumi.dat[,2:3]) -> LumiData
>  rownames(LumiData) <- Lumi.dat[,1]
> attach(LumiData)
> Lumi2
 [1] 1 0 1 1 0 1 1 1 1 0 1 0 0 0 0 0 1 0 0 0 1 0 0 1 0 1 0 0
> names(Lumi2) <- rownames(LumiData)
> name.check(172.tre, LumiData) -> LumiOverlap
> read.tree ("172.tre") -> tree1
> plot (tree1)
> name.check(tree1, LumiData) -> LumiOverlap
> plot (LumiOverlap)
> name.check(tree1, LumiData)
```

```
[1] "OK"
> Lumi2Label <- character(length(tree1$tip.label))
> names(Lumi2Label) <- names(Lumi2)
> Lumi2Label[Lumi2==0] <- "red"
> Lumi2Label[Lumi2==1] <- "blue"
> plot(tree1, cex=0.5, label.offset=2)
> points(rep(104, length(tree1$tip.label)), 1:length(tree1$tip.label), pch=22,
bg=Lumi2Label[tree1$tip.label], cex=0.5, lwd=0.25)
> ace(Lumi2, tree1, type="discrete") -> tree1Lumi2
> tree1Lumi2

    Ancestral Character Estimation

Call: ace(x = Lumi2, phy = tree1, type = "discrete")

    Log-likelihood: -8.745127

Rate index matrix:
  0 1
0 . 1
1 1 .

Parameter estimates:
 rate index estimate std-err
          1    0.001   6e-04

Scaled likelihoods at the root (type '...$lik.anc' to get them for all nodes):
          0           1
0.994056219 0.005943781

#plotting results on timetree estimated with BEAST
> plot(tree1, show.node.label=FALSE, label.offset=3, cex=0.4)
> points(rep(105, length(tree1$tip.label)), 1:length(tree1$tip.label), pch=22,
bg=Lumi2Label[tree1$tip.label], cex=0.5, lwd=0.25)
> nodelabels(pie=tree1Lumi2$lik.anc, cex=0.5)
```

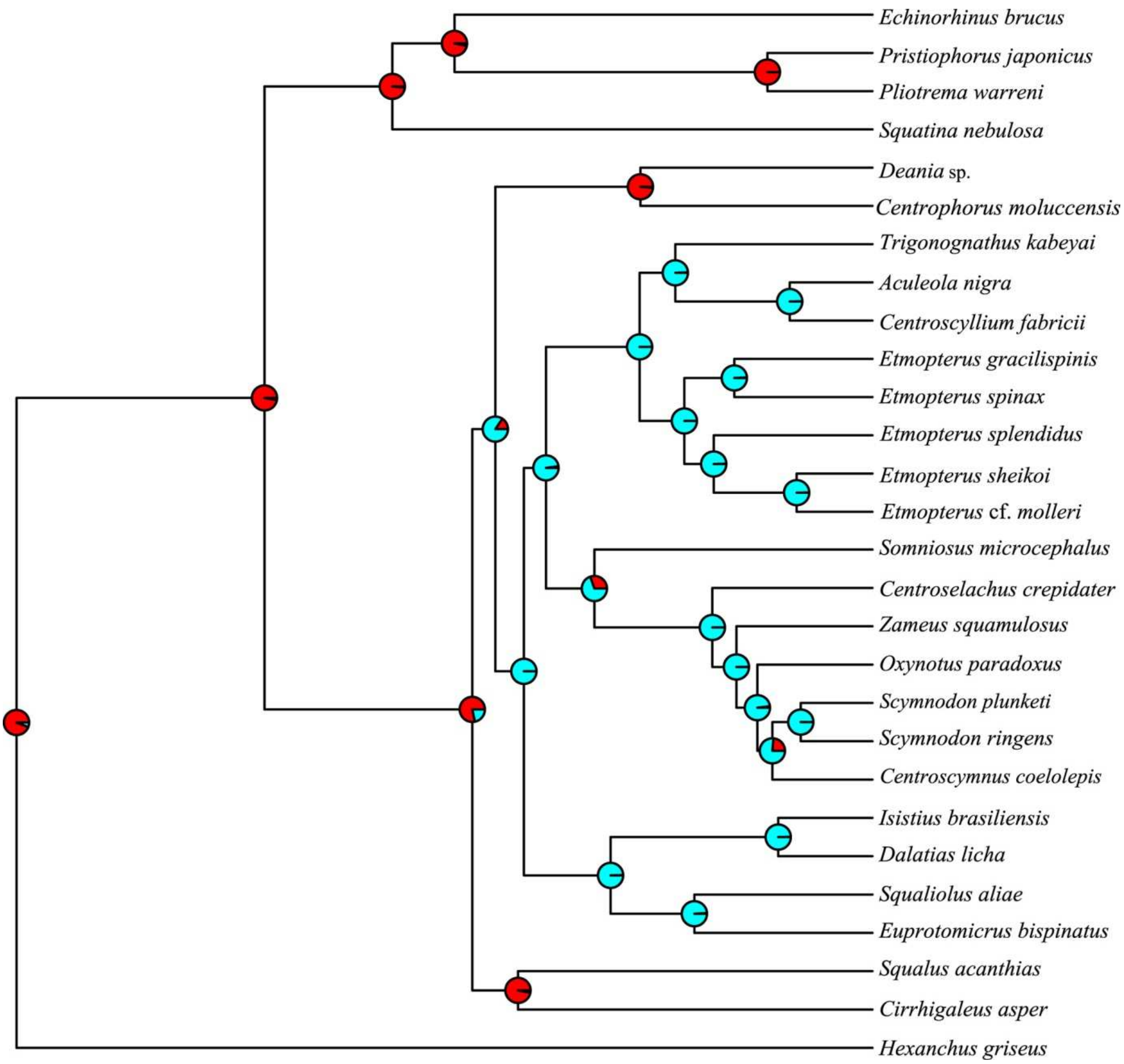

Figure S9: ancestral bioluminescence of Squaliformes. Pie charts indicate the probability that ancestral taxa are luminescent (blue) or not (red). Families Etmopteridae and Dalatiidae were coded as luminous as well as genera *Centroselachus*, *Centroscymnus*, *Scymnodon*, and *Zameus* within Somniosidae referring to [8].
